# Molecular and Cellular Characterization of an AT-Hook Protein from *Leishmania*

**DOI:** 10.1371/journal.pone.0021412

**Published:** 2011-06-23

**Authors:** Ben L. Kelly, Gyanendra Singh, Ashok Aiyar

**Affiliations:** 1 Department of Microbiology, Immunology and Parasitology, Lousiana State University Health Sciences Center, New Orleans, Louisiana, United States of America; 2 Stanley S. Scott Cancer Center, Lousiana State University Health Sciences Center, New Orleans, Louisiana, United States of America; University of South Alabama, United States of America

## Abstract

AT-rich DNA, and the proteins that bind it (AT-hook proteins), modulate chromosome structure and function in most eukaryotes. Unlike other trypanosomatids, the genome of *Leishmania* species is unusually GC-rich, and the regulation of *Leishmania* chromosome structure, replication, partitioning is not fully understood. Because AT-hook proteins modulate these functions in other eukaryotes, we examined whether AT-hook proteins are encoded in the *Leishmania* genome, to test their potential functions. Several *Leishmania* ORFs predicted to be AT-hook proteins were identified using *in silico* approaches based on sequences shared between eukaryotic AT-hook proteins. We have used biochemical, molecular and cellular techniques to characterize the *L. amazonensis* ortholog of the *L. major* protein LmjF06.0720, a potential AT-hook protein that is highly conserved in *Leishmania* species. Using a novel fusion between the AT-hook domain encoded by LmjF06.0720 and a herpesviral protein, we have demonstrated that LmjF06.0720 functions as an AT-hook protein in mammalian cells. Further, as observed for mammalian and viral AT-hook proteins, the AT-hook domains of LmjF06.0720 bind specific regions of condensed mammalian metaphase chromosomes, and support the licensed replication of DNA in mammalian cells. LmjF06.0720 is nuclear in *Leishmania*, and this localization is disrupted upon exposure to drugs that displace AT-hook proteins from AT-rich DNA. Coincidentally, these drugs dramatically alter the cellular physiology of *Leishmania* promastigotes. Finally, we have devised a novel peptido-mimetic agent derived from the sequence of LmjF06.0720 that blocks the proliferation of *Leishmania* promastigotes, and lowers amastigote parasitic burden in infected macrophages. Our results indicate that AT-hook proteins are critical for the normal biology of *Leishmania*. In addition, we have described a simple technique to examine the function of *Leishmania* chromatin-binding proteins in a eukaryotic context amenable to studying chromosome structure and function. Lastly, we demonstrate the therapeutic potential of compounds directed against AT-hook proteins in *Leishmania*.

## Introduction

AT-rich sequences in chromosomal DNA are closely associated with cellular processes such as DNA replication and transcription in many eukaryotes. In this context, the genomes of *Leishmania* sp. are unusual even amongst other trypanosomatids by virtue of their high GC-content, as high as 60% in *Leishmania major* [1]. Despite this GC-bias, it is apparent there is a skewed distribution of AT-rich sequences in *Leishmania* chromosomes. For example, the strand-switch regions (SSRs) from where transcription initiates [2], [3], are >50% AT-rich [4], [5].

In many eukaryotes, a class of proteins, termed AT-hook proteins, is central to DNA replication, repair and transcription [6], [7], [8], [9], [10], [11], [12], [13]. These proteins contain a motif termed the AT-hook that binds the minor groove of AT-rich DNA [14]. Their effector functions result from either inducing conformational changes in the bound sequences, or from other domains of these proteins recruiting components of the replication, repair or transcription pathways. The AT-hook motif is typically a short repeat of glycine and arginine flanked by a proline at one or both ends, for example GRGRP, PRGRP, or KRGRP [15]. The central RGR of the AT-hook inserts into the minor groove of AT-rich DNA, where there are interactions between arginine and thymidine [16], [17]. The absence of a side-chain on glycine also facilitates minor groove insertion. In addition to canonical AT-hooks, other sequences with repeats of glycine and arginine (GR repeat sequences) can also bind AT-rich DNA. Nucleolin, a nucleolar protein contains an extended GGR repeat and binds the T-strand of AT-rich DNA with high affinity [18]. Our previous studies have shown that the sequence GRGRGRGRGRGGGRP in the EBNA1 protein of Epstein-Barr virus binds AT-rich DNA with very high specificity [19].

The prototypic eukaryotic AT-hook protein is the mammalian HMGA1a protein [14]. This protein contains three AT-hooks. In mammalian cells, HMGA1a is essential to form transcription enhanceosomes [10], [11], and can recruit the licensed DNA replication machinery to chromosomal replication origins [9], [13]. Indeed, the multi-subunit replication origin recognition complex (ORC) in budding and fission yeasts also recognizes AT-rich DNA [7], [12]. In fission yeast, the Orc4 subunit of ORC contains nine AT-hooks (*ibid*), whereas Orc2 in *S. cerevisiae* contains one AT-hook [20]. Although the mammalian ORC proteins do not contain AT-hook motifs, HMGA1a was recently shown to recruit mammalian ORC to replication origins [9]. A function for AT-hooks in licensed DNA replication extends to mammalian DNA viruses that replicate as episomes in infected mammalian cells. For Epstein-Barr virus (EBV), the viral protein Epstein-Barr nuclear antigen 1 (EBNA1) uses a site-specific DNA-binding domain to bind the virus replication origin, and AT-hook domains to recruit ORC to this origin [13].

It is common for AT-hook proteins to function in multiple processes. The functional duality described above for HMGA1a is also observed for EBV's EBNA1 protein, which functions in replication and transcription activation [13], [21]. In *S. cerevisiae*, Orc2 is critical to establish transcriptional silencing at the HML and HMR mating type loci in addition to its replication function [22], [23]. Displacement of AT-hook proteins from bound sequences can dramatically alter cellular and organismal phenotypes. For instance, specific displacement of the *Drosophila* AT-hook protein, D1, from bound chromosomal AT-rich sequences using a peptidomimetic agent, or a synthetic AT-hook protein, alters patterned gene expression programs, position effect variegation, and development [24], [25], [26], [27]. These studies also indicate that cell-permeant peptidomimetics directed against specific AT-hook motifs can profoundly affect cellular function.

Because the distribution of AT-rich sequences within the GC-rich *Leishmania* genomes is localized to functional elements such as SSRs, we hypothesized that AT-hook proteins were likely to be encoded within the *Leishmania* genome. We anticipated that if such proteins were expressed in *Leishmania*, they would participate in essential processes such as transcription and DNA replication, and therefore represent novel targets for therapeutic intervention against *Leishmania*sis.

We mined the *L. major* genome to identify AT-hook proteins, and have functionally characterized the *L. amazonensis* homolog of the *L. major* protein encoded by LmjF06.0720, which we have termed LamAT-Y. We focused on LmjF06.0720 because its homologs are highly conserved in all sequenced *Leishmania* sp., but not the mammalian host, rendering it an excellent candidate target for therapeutic intervention. Our results indicate that LamAT-Y is expressed in both promastigote and amastigote stages, and that its AT-hook domains are functionally equivalent to the AT-hook domains of HMGA1a. Further, we have used the sequence of LamAT-Y to design a peptidomimetic that inhibits replication of promastigotes and intracellular amastigotes without substantive effects on mammalian cells. We discuss these results in the context of *Leishmania* molecular biology, and the rational design of parasite-selective therapies against *Leishmania*sis.

## Results

### The N-terminal AT-hook domain of the *L. major* gene LmjF06.0720 is conserved uniquely in Leishmania species

**Figure 1:**
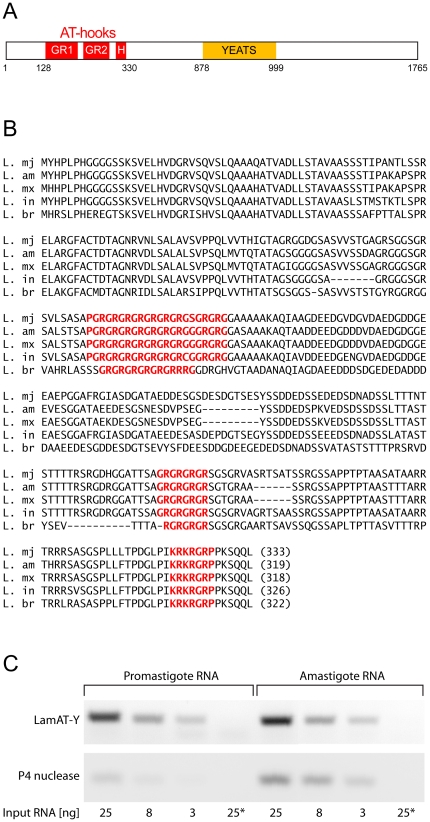
Sequence analysis and expression of the predicted N-terminal AT-hook domain of LmjF06.0720 in *L. major* and its orthologs in *L. amazonensis, L. mexicana, L. infantum*, and *L. braziliensis*. (A) Schematic of the entire open reading frame predicted by LmjF06.0720. GR indicates Glycine-Arginine dipeptide repeats; ATH denotes the classical RGRP AT-hook motif; YEATS, indicates YEATS protein family domain. (B) Sequence alignment of the first ∼330 amino-acids in LmjF06.0720 and its orthologs in *L. amazonensis*, *L. mexicana, L. infantum* and *L. braziliensis*. GR1, GR2 and ATH are emphasized in red (C) Semi-quantitative analysis of LamAT-Y expression. Total RNA was isolated from *L. amazonensis* promastigotes and axenic amastigotes and quantified prior to cDNA synthesis. The cDNAs were then amplified by PCR for 30 cycles with input amounts of 3, 8 and 25 ng RNA as denoted using LamAT-Y-specific and P4 nuclease-specific primers and analysed by agarose gel electrophoresis as indicated. Lane 25* indicates PCRs performed with RNA templates that were not reverse-transcribed.

In light of the functions of AT-hook proteins in other eukaryotes, and the AT-richness of SSRs, we employed two strategies to mine the *L. major* genome to identify candidate *Leishmania* AT-hook proteins. In the first of these, we searched for proteins that contained canonical AT-hooks such as those in HMGA1a (Table S1), whereas in the second, we used PSI-BLAST searches to identify sequences of low-complexity that contained at least three glycine-arginine (GR) or arginine-glycine (RG) dipeptide repeats (Table S2), which are also known to bind AT-rich DNA. Six proteins were identified with canonical AT-hooks, and 21 proteins with at least three GR/RG repeats. One of these 27 proteins was pursued in this study because in addition to a canonical AT-hook, this protein, LmjF06.0720, also contains two long GR repeats. LmjF06.0720 also contains a YEATS domain, which is associated with transcription regulation and chromatin remodeling [28], [29]. LmjF06.0720 is predicted to encode a 1765 amino acids (a.a.) protein and is depicted schematically in Figure 1A. The amino-terminal 330 a.a. of LmjF06.0720 contains two extended GR repeats, labeled GR1 and GR2, and one canonical AT-hook, labeled H. This protein is predicted to contain a YEATS domain from a.a. 878-999. The GR repeats and AT-hook of LmjF06.0720 are highly conserved in all *Leishmania* species (Figure 1B), including the more distantly related *L. braziliensis*, yet absent from all putatively equivalent YEATS domain-containing proteins in other trypanosomatids. A nuclear localization sequence (NLS) with the sequence LPIKRKRGRP between is predicted between a.a. 318-327 [30], [31], and contains the canonical AT-hook. This NLS is conserved in the orthologs of LmjF06.0720 in other Leishmania sp. (Figure 1B). Algorithms that predict protein sub-cellular localization indicate LmjF06.0720 to be nuclear or shuttle between the nucleus and cytoplasm [31], [32]. Structural predictions indicate the amino-terminal ∼700 a.a. to be highly solvent accessible with no predicted structural elements other than a short alpha-helix and short beta-sheet between a.a. 15-50 [31]. In contrast to the first ∼700 a.a., the rest of LmjF06.0720 is predicted to be highly structured with a large number of beta-sheets and alpha-helices predicted between a.a. ∼900 to the C-terminus (*ibid*). These predictions indicate a very high propensity for multiple alpha-helices between a.a. 1450 to the C-terminus (*ibid*). Curiously, this region is also predicted to have severely decreased solvent accessibility, perhaps indicative of it being buried with the three-dimensional structure of LmjF06.0720 or participating in hydrophobic interactions with other proteins.

In this study, we chose to focus on the *L. amazonensis* homolog of LmjF06.0720, termed LamAT-Y, because, unlike *L. major*, *L. amazonensis* can be differentiated *in vitro* into axenic amastigotes [33], [34], permitting evaluation of LamAT-Y in both promastigote and amastigote stages. In preliminary experiments, we used filter binding assays that confirmed the GR1 and ATH domains of LamAT-Y specifically bound AT-rich DNA *in vitro* (Supplementary Information Figure S1).

Next, we examined the expression of LamAT-Y in promastigotes and amastigotes. For this, mRNA extracted from promastigotes and axenic amastigotes was subjected to reverse-transcriptase PCR (RT-PCR). This analysis, shown in Figure 1C, indicated that LamAT-Y is expressed both in promastigotes and axenic amastigotes at relatively equivalent levels based on the amount of input RNA in each reaction. As a control, we examined the expression of P-4 nuclease [35], whose expression is upregulated in amastigotes (Figure 1C).

**Figure 2:**
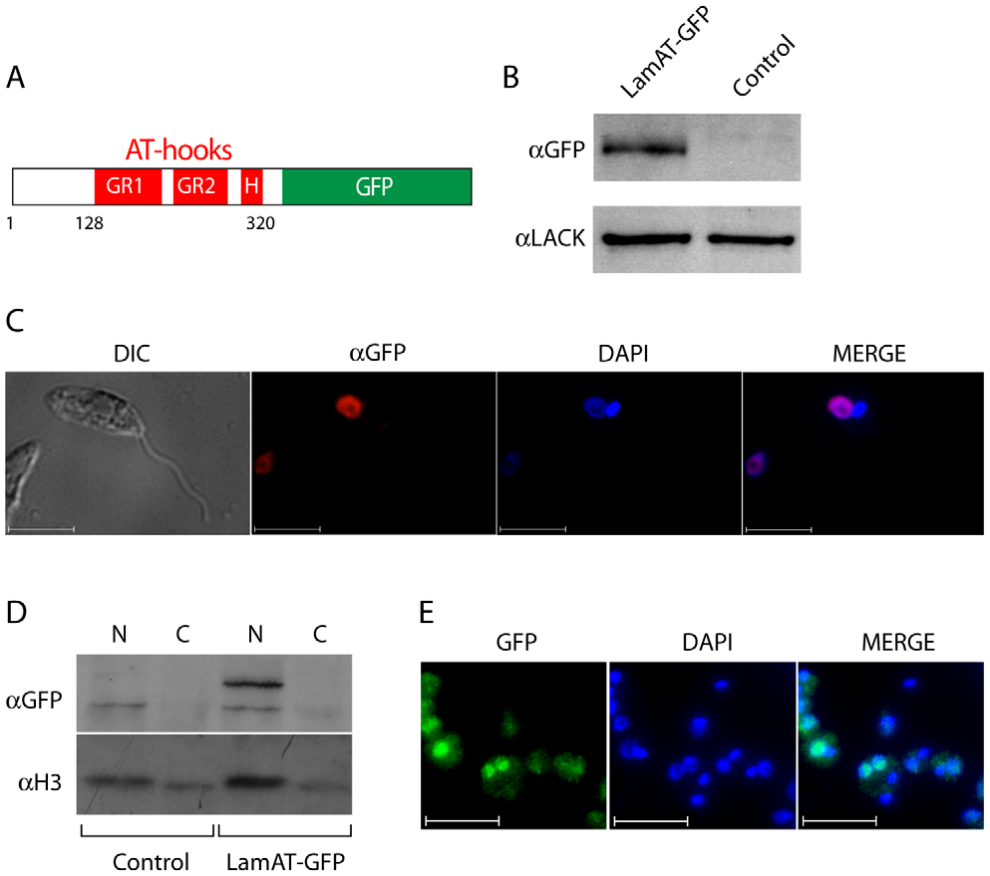
LamAT-GFP localizes to the *Leishmania* nucleus. (A) Schematic of the LamAT-GFP chimera stably expressed in *L. amazonensis*. (B) Immunoblot analysis of lysates from LamAT-GFP transfectants and wildtype controls using anti-GFP antisera ab290. Blots were stripped and probed with anti-LACK antisera as a loading control (C) Parasites were fixed, cytocentrifuged onto slides, immunostained with anti-GFP (ab290), followed by a Texas Red-conjugated secondary antibody and counterstained with DAPI. Parasites were then visualized at 630X under DIC illumination or by indirect immunofluorescence. Bars indicate a scale of 10 µm. (D) Immunoblotting was performed on nuclear and cytoplasmic extracts of control and LamAT-GFP expressing *L. amazonensis* and probed with antisera against GFP and histone H3, as indicated. (E) Visualization of axenic amastigote forms of *L. amazonensis* expressing LamAT-GFP. LamAT-GFP was visualized by direct fluorescence, and nuclei were counterstained with DAPI. Bars indicate a scale of 10 µm.

Because our attempts to generate polyclonal antisera against LamAT-Y did not yield antibodies of adequate specificity, we further characterized the AT-hooks of LamAT-Y by fusing the amino-terminal 330 a.a. of LamAT-Y to GFP to create the chimera, LamAT-GFP, depicted in Figure 2A.

### LamAT-GFP is a nuclear protein in promastigotes and amastigotes

A plasmid encoding LamAT-GFP was introduced into *L. amazonensis* and maintained under G418 selection. As indicated in Figure 2B, transfectants expressed a fusion protein of the expected size as determined using anti-GFP antisera. We examined the expression of LamAT-GFP relative to GFP at the levels of mRNA and protein. These analyses, shown in Supplementary Information Figure S2, indicated that although LamAT-GFP is expressed equivalently to GFP at the level of mRNA (Figure S2A), the level of LamAT-GFP protein is expressed at approximately 1.5% the level of GFP protein (Figure S2B). These experiments also indicated that the mRNA levels of LamAT-Y are approximately 20% the levels of LamAT-GFP (Figure S2C).

Indirect immunofluorescence, performed using anti-GFP antisera indicated that LamAT-GFP was nuclear in promastigotes (Figure 2C). Consistent with this localization, fractionation of promastigotes into nuclear and cytoplasmic fractions indicated that LamAT-GFP was predominantly localized in the nuclear fraction (Figure 2D). The localization of histone H3 was used as a control for this analysis (*ibid*). Finally, we examined the localization of LamAT-GFP in non-motile axenic amastigotes using direct fluorescence microscopy. Here also, LamAT-GFP was observed to be predominantly nuclear (Figure 2E). We did not observe LamAT-GFP localizing to kinetoplasts in either amastigotes or promastigotes (Figures 2C, 2E). These analyses confirm predictions that LamAT contains an NLS, and support the predicted nuclear localization of LamAT-Y.

### Netropsin affects promastigote replication and disrupts nuclear localization of LamAT-GFP

The actinobacterial oligopeptide netropsin binds AT-rich DNA with high specificity and affinity [36], [37], and consequently displaces AT-hook proteins from such sequences [38]. Therefore, we examined its activity against *L. amazonensis* promastigotes and determined its effects on the localization of LamAT-GFP.

**Figure 3:**
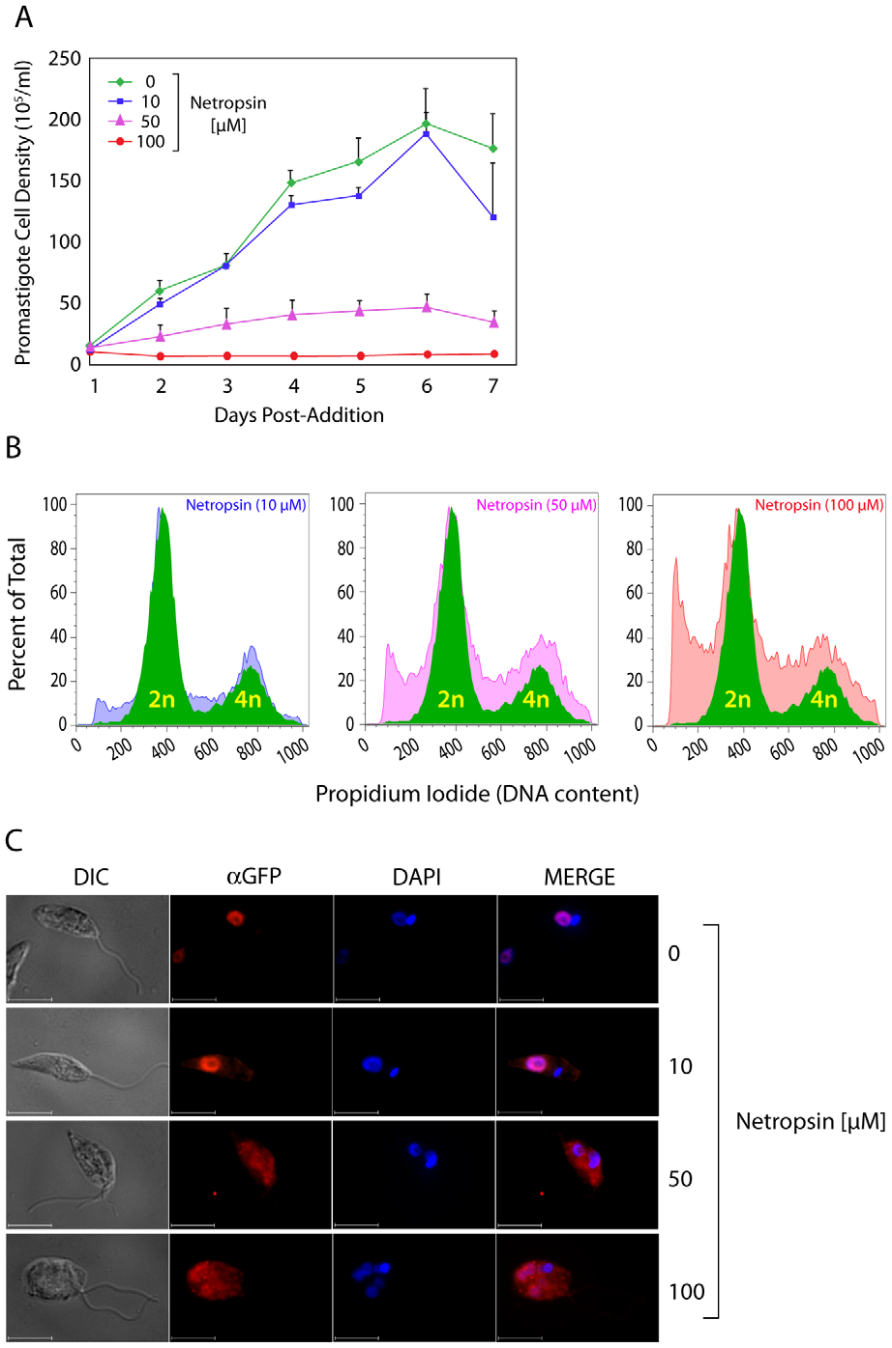
Pharmacological agents that bind the minor groove of AT-rich DNA inhibit the growth of *Leishmania* and disrupt the nuclear localization of LamAT-GFP. (A) *L. amazonensis* promastigotes were cultured in the presence of the indicated concentrations of netropsin for up to seven days. Parasite densities are indicated. (B) The effect of netropsin on promastigote DNA content. After treatment of parasite cultures for 12 hrs with the indicated concentrations of netropsin, parasites were fixed, stained with PI and analysed by flow cytometry to determine DNA content. The distribution of DNA from control untreated cells into 2N and 4N peaks is indicated by the solid green profile superimposed upon the netropsin-treated profiles. The percent of total indicates the number of positive events relative to the total number of parasites passed through the flow cytometer in that run. (C) The effect of netropsin on the localization of LamAT-GFP. Parasites that had been cultured for 48 hr in the indicated concentrations of netropsin were fixed and cytocentrifuged onto slides. LamAT-GFP was visualized by indirect immunofluorescence as described in the Methods; nuclei and kinetoplasts were counterstained with DAPI. Bars indicate scale (10 µm).

As shown in Figure 3A, netropsin interfered with the proliferation of promastigotes in a dose-dependent fashion. At low doses, such as 10 µM, netropsin had a neglible effect on proliferation for up to one-week post-addition of the drug. Higher doses of netropsin severely abrogated promastigote proliferation; these effects were detected as early as two-days post drug addition. We examined the effect of netropsin on promastigote DNA content by propidium iodide (PI) staining of fixed parasites. As early as 12 hours post-addition, netropsin affected the distribution of DNA (Figure 3B). The distribution of DNA from untreated cells into 2N and 4N peaks is indicated by the solid green profile. Treatment with 10 µM netropsin yielded a small sub-G1 population. In addition, the fraction of S-phase cells, between the 2N and 4N peaks, also increased. Finally, a small increase in the number of post-S phase 4N cells was also observed. All three effects were more pronounced with increased netropsin concentrations (*ibid*). These data indicate that netropsin interferes with completion of S-phase, and with the passage of cells from S to G2, or post-G2. We examined these effects further using direct fluorescence for DNA content and indirect immunofluorescence to localize LamAT-GFP. In this assay, parasites treated with the indicated concentrations of netropsin (Figure 3C) were fixed, immunostained for LamAT-GFP, and counterstained with DAPI. At low concentrations of netropsin (10 µM), LamAT-GFP remained predominantly nuclear, consistent with minimal effects of this netropsin level on promastigote growth (Figure 3A). At higher concentrations of netropsin, we observed large numbers of shrunken cells or debris. Double-staining using Annexin V and propidium iodide indicated that the shrunken cells/debris either resulted from necrosis or late-stage apoptosis (data not shown). In addition, about 20-50% of the promastigotes displayed an abnormal morphology. These cells were often multi-flagellated, and contained more than one nucleus. Therefore, it is likely that the increase in the 4N and >4N populations (Figure 3B) at higher netropsin concentrations is due to the accumulation of cells with two or more nuclei.

Concurrent with these morphological aberrations, we observed LamAT-GFP to be displaced from the nucleus (Figure 3C), suggesting that nuclear localization of LamAT-GFP results from the association of its AT-hooks with nuclear AT-rich DNA. We interpret the effect of netropsin on normal replication of promastigotes to indicate that netropsin affects LamAT-Y, other *Leishmania* proteins that bind AT-rich DNA, or cellular processes that require AT-rich DNA.

It is likely that LamAT-Y is essential for *Leishmania* because our attempts to make even a haploid knockout were unsuccessful. Because the absence of antibodies against intact LamAT-Y was a further technical impediment to dissect the function of this protein in *Leishmania*, we chose to do so in mammalian cells using two well-described properties of HMGA1a and other AT-hook proteins. The rationale for this approach was based on prior observations that over-expression of either *Leishmania* or mammalian histone H1 have indistinguishable effects in mammalian cells [39], [40]. Unlike the other histones, but akin to AT-hook proteins, histone H1 preferentially associates with AT-rich DNA [41], [42], [43]. For this reason, we think it is likely that properties displayed by LamAT-Y in mammalian cells provide some indication of this protein's properties in *Leishmania*.

### LamAT confers nuclear localization and punctate mitotic chromosome association in mammalian cells

**Figure 4:**
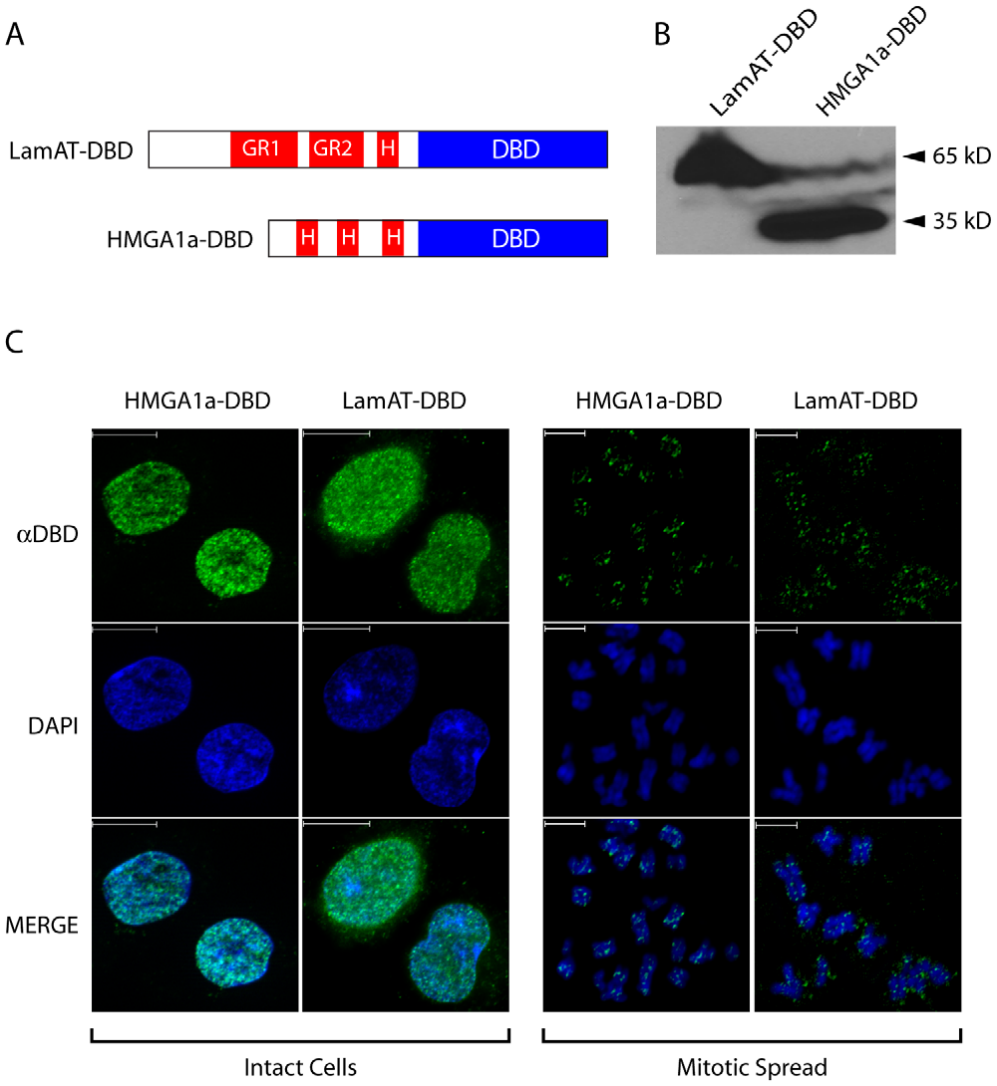
LamAT associates with eukaryote chromosomes. (A) Schematic representation of LamAT (LamAT-DBD) or HMGA1a (HMGA1a-DBD) stably expressed in 293 cells as fusions with the DNA-binding domain of EBNA1. (B) Immunoblots of 293 cell lysates expressing LamAT-DBD or HMGA1a-DBD visualized using anti-DBD antisera. (C) Localization of LamAT-DBD and HMGA1a-DBD in intact 293 cells (left panels) or on mitotic chromosomes (right panels) visualized by indirect immunofluorescence using anti-DBD antisera. DNA was counterstained with DAPI.

We characterized the localization and mitotic chromosome binding properties of LamAT (Figure 4) using a chimeric protein containing the DNA binding domain (DBD) of the EBV EBNA1 protein to facilitate further functional studies (Figure 4). Chimeric proteins, LamAT-DBD, or HMGA1a-DBD, depicted in Figure 4A, were stably expressed in human 293 cells. As shown in Figure 4B, both chimeras were expressed at equivalent levels and were of the expected size. Although it lacks a distinct nuclear localization sequence (NLS), HMGA1a localizes to the nucleus, a property observed previously for HMGA1a-DBD by others and us. As detected by indirect immunofluorescence of intact cells using a primary antibody against DBD, the LamAT-DBD chimera was nuclear (Figure 4C). Studies by the groups of Reeves, Laemmli and others have shown that HMGA1a and other AT-hook proteins bind eukaryotic mitotic chromosomes with a characteristic punctate pattern [44], [45]. This pattern arises from the distribution of the AT-queue of AT-rich sequences along a chromosome, such that some of these sequences are in uncondensed loops emanating from the chromosome body that are easily accessed by AT-hook proteins [45], [46]. Because these AT-rich loops stain poorly with DAPI, AT-hook proteins are observed as punctate dots slightly removed from the body of the chromosome [19], [44], [47]. We observed this localization for LamAT-DBD and HMGA1a-DBD in mitotic chromosome spreads from 293 cells stalled in mitosis using colcemid (Figure 4C). Our previously published studies demonstrate that DBD alone does not associate with mitotic chromosomes [19], [47]. These data show that LamAT confers punctate mitotic chromosome association to DBD, and this localization is indistinguishable from that of a *bona fide* AT-hook protein (HMGA1a) in mammalian cells. Next, we used a well-characterized assay for the replication and mitotic partitioning of a stable episomal plasmid in mammalian cells [48], [49], [50], used previously for HMGA1a [47], to functionally characterize LamAT.

### LamAT-DBD mediates retention, replication and partitioning of an episomal plasmid in mammalian cells

The episomal nuclear genome of Epstein-Barr virus (EBV) undergoes licensed replication and stable partitioning in EBV-immortalized cells. A single viral protein, termed EBNA1, and a sequence within the EBV genome, termed *oriP*, which is bound by EBNA1, confers both these properties upon the EBV episome. EBNA1 possesses a C-terminal site-specific DNA binding domain (DBD) [51], which specifically associates with two sets of sequences within *oriP*, termed the family of repeats (FR) and the dyad symmetry element (DS) [52], [53]. The amino-terminal 370 a.a of EBNA1 contains multiple glycine-arginine (GR) repeats that closely resemble the repeats in LamAT, and function as AT-hooks [19]. Previous studies indicate that HMGA1a, or other proteins that preferentially bind AT-rich DNA, but not proteins that non-specifically bind chromosomal DNA, can functionally substitute for the amino-terminal AT-hooks of EBNA1 [47]. In addition to chromosome binding, the AT-hooks of EBNA1 and HMGA1a can recruit the cellular origin recognition complex (ORC) to facilitate licensed replication of EBV episomes or other plasmids in mammalian cells [13]. These observations led us to test whether LamAT could functionally substitute for the AT-hooks of EBNA1 using well-established plasmid maintenance assays conducted in human cells.

**Figure 5:**
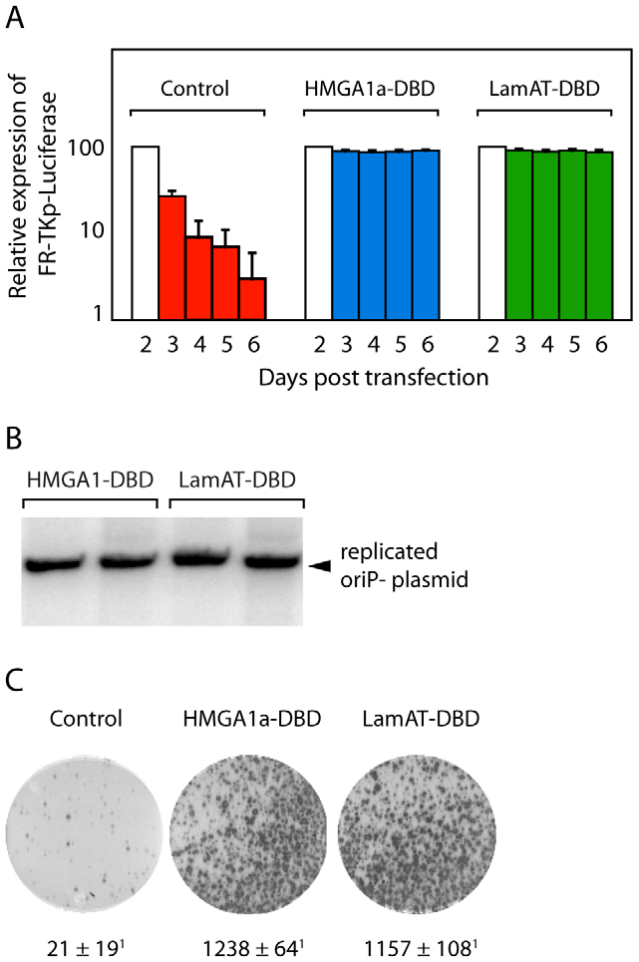
LamAT-DBD confers stability, replication and partitioning to plasmids that contain EBV elements bound by DBD. (A) 293 cells or 293 cells stably expressing either LamAT-DBD or HMGA1a-DBD were transfected with FR-containing luciferase reporter plasmid, following which luciferase activity was monitored from 48-144 hr post-transfection as described in the Methods. (B) 293 cells stably expressing LamAT-DBD or HMGA1a-DBD were transfected with a puromycin-expressing replication reporter plasmid that contains EBV's *oriP* element. Plasmid DNA replication was measured as a function of resistance to digestion by *Dpn*I as described in the Methods. (C) Partitioning of the puromycin-containing replication reporter plasmid used in B was assayed as described in the Methods. 10^5^ live-transfected 293 cells were selected with puromycin for 3 weeks post plating, following which colonies were stained by methylene blue and counted with ImageJ as described previously [19]. The control cells expressed DBD alone.

Unlike protist eukaryotes in which mitosis is not accompanied by dissolution of the nuclear membrane, transfected plasmids are inherently unstable in mammalian cells. This is illustrated by the fate of transfected origin-containing plasmids in yeast versus mammalian cells. In yeast, these plasmids are maintained as episomes under selection [54]. In contrast, origin-containing plasmids are unstable in mammalian cells [55], [56], [57]. Some mammalian viruses, such as EBV, have developed strategies to permit maintenance of their genomes as nuclear episomes. EBV's genome contains a cis-acting sequence, FR, which permits episome maintenance, when bound by EBNA1 or HMGA1a-DBD [47], [55], [57]. Therefore, we tested whether LamAT-DBD conferred stability to an FR-containing plasmid as a measure of its AT-hook function. In this experiment, shown in Figure 5A, an FR-containing plasmid that expresses luciferase was transfected into either control 293 cells, or derivatives of these cells that stably express HMGA1a-DBD or LamAT-DBD. Both luciferase mRNA [58], and enzymatic activity [59], [60] have a short half-life; therefore, the amount of measured luciferase activity over-time is proportional to the amount of episomal template remaining in transfected cells. As seen in Figure 5A, in control 293 cells, the FR-containing reporter plasmid is lost at a rate close to the dilution occurring via cell-division. In contrast, for 293 cells stably expressing HMGA1a-DBD or LamAT-DBD, this reporter was stable for the duration of the experiment, as indicated by similar levels of expression maintained for at least one week post-transfection.

EBNA1 and HMGA1a-DBD have been shown to support stable replication of *oriP*-plasmids by recruiting ORC [13]. Therefore, we tested whether LamAT-DBD would also support the replication of a transfected *oriP*-plasmid. In this assay, the *oriP*-plasmid was isolated from a dam+ *E. coli* strain, and is consequently susceptible to digestion by the restriction endonuclease *Dpn*I [56]. Mammalian cells are unable to methylate adenosines, and therefore the replication of transfected plasmids in mammalian cells results in the generation of *Dpn*I-resistant daughter plasmids [56], [61]. When evaluated three weeks post-transfection, LamAT-DBD supported the episomal replication of an *oriP*-plasmid as efficiently as HMGA1a-DBD (Figure 5B).

It is known that *oriP*-plasmids are partitioned non-randomly, such that in approximately 80-95% of all mitoses, the daughter plasmids resulting from each parental plasmid are partitioned equally into the two daughter cells [62], [63]. The efficiency of this distribution can be measured using a colony formation assay [47], [48], [49], [50]. In these assays, cells transfected with an *oriP*-plasmid that also bears a drug-resistance marker are plated as single cells and evaluated for their capacity to form a puromycin-resistant colony after three weeks. Previous experiments have established that HMGA1a-DBD partitions such plasmids indistinguishably from EBNA1, and approximately 3-logs over the partitioning efficiency of DBD alone [19], [47]. As shown in Figure 5C, LamAT-DBD also supported colony formation to the same extent as HMGA1a-DBD, and approximately 3-logs over background levels of colonies formed by random chromosomal insertion of the transfected plasmid. The experiments shown in Figure 5 reiterate that LamAT behaves similarly to HMGA1a in these cellular assays for AT-hook function.

### A novel peptidomimetic antagonizes LamAT function in mammalian cells and interferes with *Leishmania* replication

**Figure 6:**
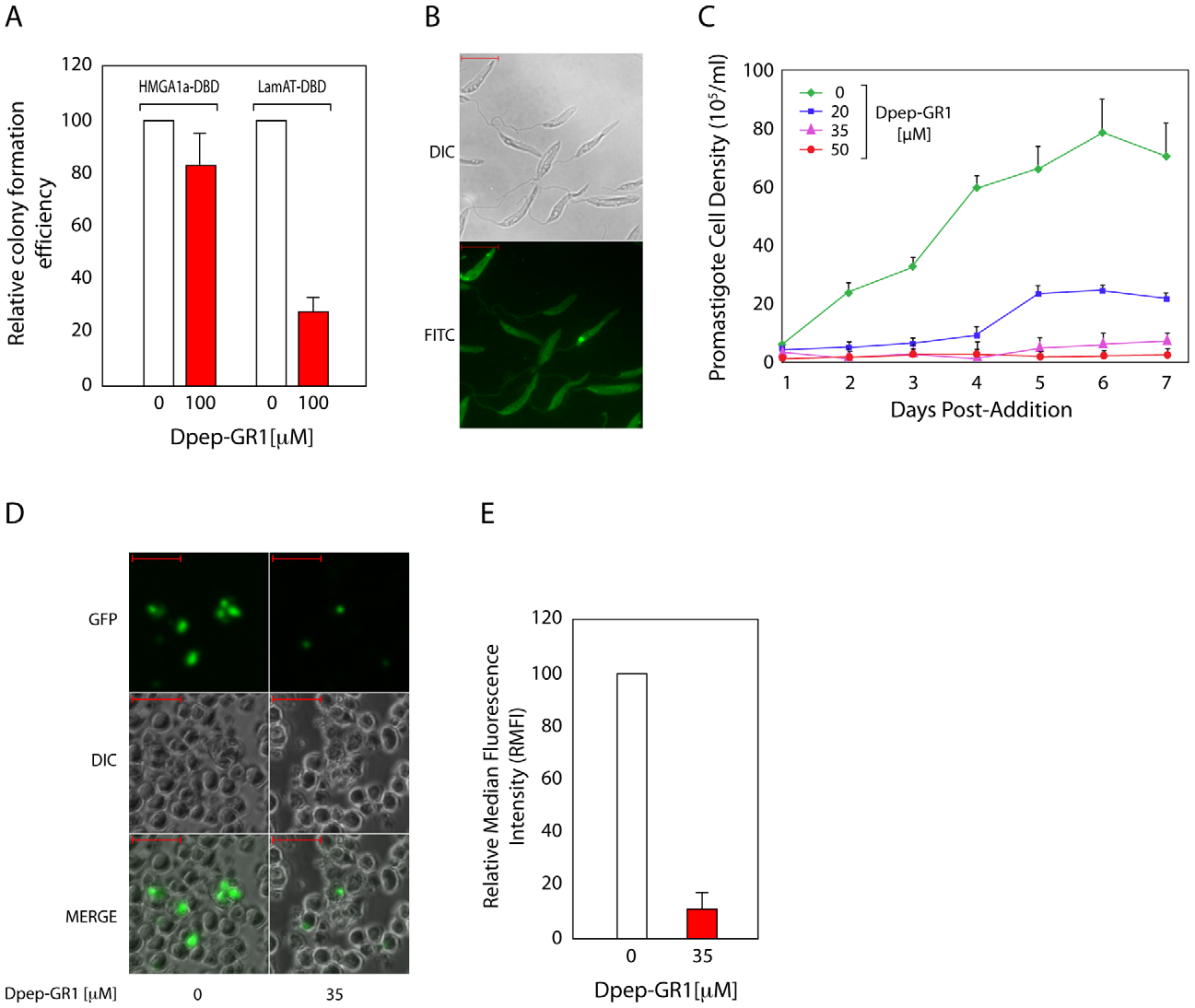
Treatment of infected macrophages with the Dpep-GR1 peptidomimetic interferes with LamAT-DBD function and concurrently reduced parasite burden. (A) 293 cells stably expressing either LamAT-DBD or HMGA1a-DBD were transfected with the replication reporter plasmid described in Figure 5. Transfected cells were treated with 100 µM Dpep-GR1 for two days after plating, and puromycin-resistant colonies formed were enumerated after 3 weeks. (B) Dpep-GR1 readily enters *L. amazonensis* promastigotes. Promastigotes were treated with 1 µM of FITC-labelled peptide Dpep-GR1 for one hr, washed twice in PBS, cytocentrifuged onto slides and visualized by direct fluorescence. Bars indicate scale (10 µm). (C) *L. amazonensis* promastigotes were cultures in the presence of the indicated concentrations of Dpep-GR1 for up to 7 days. Cells were enumerated daily. (D) Dpep-GR1 reduces parasite burden in infected macrophages. RAW264.7 cells were infected with a *L. amazonensis* strain that expresses GFP. Cells were cultured for 12 days in the presence of Dpep-GR1 at the indicated concentrations and monitored by direct fluorescence and light microscopy. (E) Infected RAW264.7 macrophages described in D were evaluated by flow cytometry for parasite burden by gating for live GFP-positive cells at 12 days post-transfection. The relative median fluorescence intensity (RMFI) represents the MFI of the treated culture as a percent of the untreated culture's MFI.

Although peptides such as netropsin are broadly effective against AT-hook proteins, peptidomimetics have been designed that are effective against a smaller subset of AT-hook proteins by displacing them from bound sequences [24], [25]. These agents target specific AT-hook proteins based on the number and distribution of AT-hooks within the protein, which in turn dictate the sequence bound by the protein [64]. The first GR repeat (GR1, Figure 1A) in the LmjF06.0720/LamAT-Y ortholog from *L. mexicana* (a very close relative of *L. amazonensis*, whose genome has been sequenced) is unique to this ortholog when compared to all predicted *L. mexicana* proteins. Therefore, we designed a D-peptide based on the sequence of GR1 (Dpep-GR1), specifically corresponding to a.a 129-146 of LamAT-Y. Because this peptide lacks L-isomer arginine residues, it cannot be degraded in living cells. Dpep-GR1 readily entered both mammalian cells and *Leishmania* (Figure 6B) consistent with the function of similar arginine-containing peptides acting as membrane permeable protein transduction domains [65], [66]. The relative specificity of Dpep-GR1 was tested by examining its ability to disrupt *oriP*-plasmid partitioning in mammalian cells mediated by HMGA1a-DBD and LamAT-DBD. For this, transfected 293 cells were exposed to 100 µM Dpep-GR1 for two days post-transfection, prior to being assayed for colony formation. In these assays, Dpep-GR1 dramatically reduced the ability of LamAT-DBD to support colony formation, with a minimal effect on HMGA1a-DBD (Figure 6A). Therefore, at least in mammalian cells, Dpep-GR1 is selective for LamAT over HMGA1a.

Akin to netropsin, Dpep-GR1 impacted the proliferation of *Leishmania* promastigotes in a dose-dependent manner (Figure 6C), which we interpret to indicate that LamAT-Y or another *Leishmania* AT-hook protein/s is required for promastigote proliferation. We also tested the effects of Dpep-GR1 on the replication of intracellular amastigotes. RAW264.7 mouse macrophages were infected with GFP-expressing *L. amazonensis* promastigotes and passaged 24 hours post-infection, such that one half of the cells were exposed to 35 µM Dpep-GR1 for 12 days. At this concentration, Dpep-GR1 does not significantly affect macrophage survival (data not shown, and Table S3). Infected RAW264.7 cells were visualized by fluorescence microscopy, the results or which are shown in Figure 6D. We observed a small decrease in the percent of GFP-positive cells in the presence of Dpep-GR1 (Figure 6D), and quantified in Table S3. However, the fluorescence per cell decreased strikingly (Figure 6D). We quantified this decrease by measuring changes in relative median fluorescence intensity (RMFI) after in the presence or absence of Dpep-GR1. As seen in Figure 6E, there was a sharp decrease in RMFI when infected cells were exposed to Dpep-GR1 for 12 days. There are three possible explanations for the sharp decrease in fluorescence per cell: 1) That Dpep-GR1 selectively kills infected macrophages but not uninfected macrophages; 2) That Dpep-GR1 affects the expression of GFP within intracellular amastigotes; or 3) That Dpep-GR1 reduces replication of intracellular amastigotes, which in turn reduces the fluorescence per cell.

Although we cannot prove that LamAT-Y is the target of Dpep-GR1 in *Leishmania*, our data suggest that effective anti-*Leishmania*ls can be designed by targeting *Leishmania* AT-hook proteins.

## Discussion

While AT-hook proteins are known to have critical roles in both eukaryote and prokaryote gene expression and DNA replication, their function has not been studied previously in *Leishmania*. We have identified several putative AT-hook proteins encoded in the *L. major* genome, and focused on LmjF06.0720, which is predicted to contain three AT-hooks and a YEATS domain. Based on the known functions of these domains, it is predicted that LmjF06.0720 functions in transcription, DNA replication and/or chromatin modification. We analysed the *L. amazonensis* ortholog of LmjF06.0720, termed LamAT-Y, because *L. amazonensis* promastigotes can be differentiated into axenic amastigotes *in vitro*. The predicted amino acid sequence of LmjF06.0720 is conserved in its orthologs found in other sequenced *Leishmania* sp. While LmjF06.0720 is conserved in all *Leishmania* sp., potential orthologs are absent in other sequenced trypanosomatid and mammalian genomes. YEATS domains are found in proteins that modulate chromatin structure and function [28], [29]. By possessing AT-hooks and a YEATS domain, LmjF06.0720 is likely to modulate chromatin structure at AT-rich chromosomal sequences. We note that such sequences are present in the SSRs that punctuate ORF clusters in all *Leishmania* chromosomes and are considered to represent sites for transcription initiation [2], [3], [4].

We have observed LamAT-Y to be expressed in promastigotes and axenic amastigotes, and have dissected the function of the AT-hook-containing amino-terminal domain of this protein, termed LamAT. Consistent with nuclear localization, levels of a LamAT-GFP chimera are greatly enriched in nuclear compared to cytosolic extracts. Indirect immunofluorescence analysis of *L. amazonensis* transfectants expressing LamAT-GFP confirmed nuclear localization properties for LamAT. Further, exposure to netropsin, a drug that specifically displaces AT-hook domains from AT-rich DNA, disrupted the nuclear localization of LamAT-GFP, suggesting that its nuclear localization is dependent on AT-hook function. Concurrent with the nuclear displacement of LamAT-GFP and presumably other *Leishmania* AT-hook proteins, netropsin showed dose-dependent inhibitory effects on parasite cell division. Although the kinetoplast genome is highly AT-rich, we did not detect LamAT-GFP in promastigote or amastigote kinetoplasts. However, other eukaryotic AT-hook proteins are known to shuttle between the mitochondria and the nucleus [67], and our data do not exclude the possibility that LamAT-Y can do so under certain conditions.

We have used well-characterized assays in mammalian cells to examine AT-hook dependent properties of LamAT, because techniques such as examining protein association with metaphase chromosomes are unavailable in *Leishmania*. For these studies, we have exploited the domain structure of EBNA1, a herpesviral protein that requires AT-hooks for its functions [19]. EBNA1 contains N-terminal AT-hook domains, and a C-terminal DNA binding domain (DBD) that associates with cognate binding sites on the EBV genome (*ibid*). Upon binding these sites, EBNA1 supports the licensed replication and partitioning of EBV episomes in mammalian cells. We, and others, have shown that these functions require the AT-hook domains of EBNA1 [13], [19]. Previous studies indicate that the N-terminal AT-hook containing domain of EBNA1 can be functionally substituted by AT-hook proteins such as HMGA1a, but not other chromatin-binding or nuclear proteins [47]. Therefore, we have compared the function of a LamAT-DBD chimera to the previously characterized HMGA1a-DBD chimera. Like HMGA1a-DBD (and native HMGA1a), LamAT-DBD associates with mitotic chromosomes in a punctate manner. For HMGA1a, it is proposed that such punctate binding reflects an association with non-condensed AT-rich sequences in metaphase chromosomes [45], [46]. Our findings are consistent with LamAT also binding such sequences.

In addition to AT-rich DNA, AT-hooks can bind RNA [68], and more specifically G-quadruplex RNA [13]. This latter property is necessary for EBNA1, or HMGA1a, to recruit the cellular origin-binding complex (ORC) to EBV's licensed DNA replication origin (*ibid*). Like EBNA1 or HMGA1a, the Orc1 subunit of ORC also binds G-quadruplex RNA, and therefore it is posited that ORC is recruited to EBV's origin via a G-quadruplex RNA intermediate (*ibid*). Because we have observed LamAT-DBD to support stable replication of EBV origin plasmids as efficiently as HMGA1a-DBD, it is likely that LamAT retains the capacity to recruit ORC by binding G-quadruplex RNA. LamAT's capacity to bind G-quadruplex RNA is potentially very important in *Leishmania* because many *Leishmania* transcripts are highly GC-rich and contain G runs.

We have used a plasmid replication assay in mammalian cells to evaluate the function of a peptidomimetic based on the sequence of the longest GR-repeat in LamAT. This peptidomimetic, Dpep-GR1, is indicated to be relatively specific because it interferes with the function of LamAT-DBD, but not HMGA1a-DBD. Therefore, it is possible that the length of the GR-repeat within an AT-hook changes the repertoire of the bound AT-rich DNA RNA sequences. A similar conclusion was drawn from the function of synthetic proteins containing multiple AT-hooks. Proteins with 10 and 11 AT-hooks, MATH10 and MATH11, had substantially different effects on chromosome condensation than a protein with 20 AT-hooks, MATH20 [69]. Concordant with this, MATH20, but not MATH11, suppressed position effect vareigation when expressed in *Drosophila* [26]. This latter effect has been ascribed to MATH20 displacing the naturally occuring *Drosophila* protein D1, which also contains multiple AT-hooks, from bound sites [27]. Because Dpep-GR1 showed specificity toward LamAT, we tested its effects on the growth and proliferation of *L. amazonensis* promastigotes and intracellular amastigotes. Dpep-GR1 severely reduced the replication of *Leishmania* promastigotes, and reduced replication of intracellular amastigotes or decreased gene expression in them. Our findings indicate that AT-hook proteins are valid therapeutic targets for *Leishmania*sis, and that parasite-selective drugs can be developed against such proteins.

The chromosomal sequences to which LamAT-Y and other *Leishmania* AT-hook proteins are bound are unknown. Although the *Leishmania* genome is highly GC-rich, there is a skewed distribution of AT-rich sequences in regions such as the strand-switch regions (SSRs) [4], [5] from which transcription initiates [2], [3]. An example of this is seen in the SSR from chromosome 1 that has been sequenced in 15 *Leishmania* species [5]. A sequence analysis of this SSR examining its AT-richness and the distribution of uninterrupted AT-tracts is shown in Figure S3 and Table S4. Overall, this SSR is approximately 50% AT-rich, relative to the 40% AT-richness observed of the entire Leishmania genome. This SSR contains an open reading frame embedded within it (indicated as a green arrow in Figure S3), which is flanked by short AT-rich regions indicated by broken or solid red bars in the figure. Notably, these AT-rich regions are conserved in all 15 SSRs. A statistical analysis examining the distribution of uninterrupted AT-tracts within the sequence denoted by the solid red bar in Figure S3 is shown in Table S4. As seen in the table, this sequence contains a significantly greater number of uninterrupted AT-tracts of lengths between 3-9 nucleotides when compared to 30 shuffled permutations of the same sequence. A similar distribution was observed for the AT-rich region denoted by the broken red bar (data not shown). Therefore, in addition to being AT-rich, *Leishmania* SSRs are also enriched for uninterrupted AT-tracts. Notably, the lengths of these tracts coincide with the length of AT-rich sequences bound by mammalian AT-hook proteins [68], [70], consistent with the hypothesis that they are bound by Leishmania AT-hook proteins.

Some eukaryotic AT-hook proteins displace histone H1 from repressed chromatin, resulting in derepressed chromatin that is transcriptionally active [42], [43], [71], [72]. Hallmarks of derepressed chromatin include acetylated histones, particularly H3 and H4. Because overexpression of histone H1 is repressive in *Leishmania* [39], [40], and YEATS domains associate with acetylated histone H3 and H4 to promote gene expression [28], [29], [73], it is possible that LamAT-Y uses its AT-hook domains to displace repressive histone H1 whilst promoting gene expression through interactions with acetylated H3 and H4. In addition to their function in transcription, mammalian AT-hook proteins modulate RNA splicing, licensed DNA replication, and participate in DNA repair and recombination. Future studies will indicate whether AT-hook proteins are involved in these critical processes in *Leishmania*.

In summary, our findings highlight an important role for AT-hook proteins in *Leishmania* biology that may involve multiple processes including gene expression, DNA replication and DNA repair. As such, they provide impetus for future studies such as chromatin immunoprecipitation to identify sites bound by LamAT-Y and other *Leishmania* AT-hook proteins as a prelude to elucidating their functions.

## Methods

### PCR amplification, plasmid constructs, DNA sequencing and gene expression analysis

The coding sequence for LamAT-Y (5265 bp) was amplified from *L.* amazonensis genomic DNA (Text S1). Primers were chosen based on the sequence of *L. major* LmjF06.0720 that was obtained from Genbank (Accession number XM_001680791). A 934 bp sequence from the 5′ end of LamAT-Y coding sequence, corresponding to the first 331 amino-acids, was amplified from the full-length clone. This fragment, termed LamAT, and included *Bam*HI and *Bst*XI recognition sites at 5′ and 3′ ends respectively. LamAT was used to replace the HMGA1a coding sequence in the mammalian expression plasmid AGP177 [47], to create the LamAT-DBD expression plasmid. A LamAT-GFP chimera was created by ligating amplified LamAT, flanked by *Bam*HI and *Pac*I recognition sites, to codon optimized GFP from pXG-GFP+. The ligated chimera was cloned into pXG [76] to create pXG-LamAT-GFP. Total RNA was isolated from promastigote and axenic amastigote forms of *L. amazonensis* using TRIzol reagent (Invitrogen) according to manufacturer's instructions. After quantification, RNA samples were treated with DNAse (TURBO DNase, Ambion/Applied Biosystems) according to manufacturer’s protocols then used to template cDNA synthesis using a DyNAmo cDNA synthesis kit (Finnzymes/New England Biolabs) according to manufacturer’s directions. cDNAs were amplified from RNA inputs using primers specific for LamAT-Y and the amastigote-specific P-4 nuclease [35].

For quantitative real-time PCR, RNA was extracted from parasites using the Aurum total RNA extraction system (BioRad), following which 5 µM of RNA was reverse-transcribed using the iScript cDNA synthesis kit (BioRad). A series of real-time PCRs performed using the SsoFAST-EvaGreen system (BioRad), corresponding to an input of 1, 0.25, 0.0625, and 0.0156 µM of total RNA. PCRs were performed in a BioRad CFX384 cycler, and CFX Manager software was used for data analysis. The accuracy of PCR was validated by gel electrophoresis of the amplified products, and thermal denaturation curves. The amplification conditions used provided a correlation coefficient for amplification of >0.99. Ct values were determined automatically using CFX manager. Gene expression was quantified using the ΔCt method of analysis. The following primers were used for real-time PCR: GFP-5′ (TCTGCACCACCGGCAAGCTG); GFP-3′ (TGGTCGGGGTAGCGGCTGAA); LACK-5′ (AGTTCCTGCGCGACAGCCAC); LACK-3′ (AGTTCCTGCGCGACAGCCAC).

### *Leishmania* culture and transfections

*L. amazonensis* promastigotes (strain IFLA/BR/67/PH8), kindly provided by David L. Sacks, were maintained at 27°C in media 199 and antibiotics as previously described [74]. Promastigotes were transfected with 10 µg of pXGLamAT-GFP or pXG-GFP+ and initially selected in 20 µg/ml G418 as previously described [75], [76]. Cultures of drug resistant parasites were then further expanded by 1∶20 dilutions every five days. G418 concentrations were doubled at every passage up to a concentration of 300 µg/ml. Axenic amastigotes were cultured using conditions described previously [34].

### Filter binding assays

Filter binding assays were conducted using peptides corresponding to amino-acids 121-155 of LamAT-Y (SALSTSAPGRGRGRGRGRGRGRGGGRGRGGASAAA), and 290-323 (SASGSPLLFTPDGLPIKRKRGRPPKSQQLALSNT). The synthesized peptides were acetylated at the amino-terminus and amidated at the carboxy-terminus (Peptide 2.0, Chantilly, VA). Assays were conducted as described previously [19]. Briefly, 100 ng of ^32^P-labeled poly(dA-dT) was used as probe in 25 µl binding reactions that included 1 µg of poly(dA-dC)(dG-dT) as competitor and 10 pmol of the indicated peptide. Reaction volumes were raised to 1 ml after 25 minutes at room temperature and filtered through 0.1 µm Whatman nitrocellulose membranes. Filters were washed thrice, dried and analyzed by scintillation counting. Unlabeled poly(dG-dC), poly(dA-dT) and netropsin were used as competitors. Probes and competitors were purchased from Sigma.

### Netropsin and AT-hook peptidomimetic treatment of *L. amazonensis*

For promastigote growth curves, *L. amazonensis* promastigotes were cultured at a starting density of 5×10^5^ cells/ml under previously established conditions for up to one week. At the time of inoculation, netropsin (Sigma-Aldrich) was added at a final concentration of 10, 50 or 100 µM. Aliquots of cells were counted daily. For indirect immunofluorescence, *L. amazonensis* promastigotes grown to a density of 5×10^6^ cells/ml were treated with the same concentrations of netropsin detailed above for 48 hours. At this timepoint, the localization of LamAT-GFP was evaluated by indirect immunofluorescence as described later in this section. Cell morphology was evaluated by differential interference contrast microscopy. A peptide corresponding to amino-acids 133–147 of LamAT was synthesized with D-amino-acids (Peptide 2.0 Inc., Chantilly, VA). For some experiments, the peptide was C-terminally conjugated to FITC. Peptide was incubated with *L. amazonensis* promastigotes at concentrations 20, 35 or 50 µM, and growth was monitored as described above. Peptide uptake was visualized by direct fluorescence using the FITC-labeled derivative. The effect of the peptide on amastigote infections of macrophages was conducted as described below.

### Mammalian cell culture and transfections

293 cells (ATCC number CRL-1573) and RAW264.7 mouse macrophage-like cells (ATCC Number TIB-71) were cultured as described previously [47], [77]. Derivatives of 293 cells were constructed by infection with retroviral vectors expressing the HMGA1a-DBD or LamAT-DBD chimeric proteins as described previously [19], [47]. Clones were obtained by G418 selection, and pooled prior to use. Mammalian cells were transfected as described previously [49].

### Macrophage infections

RAW264.7 macrophages were seeded at a density of 2×10^5^/ml and infected at a multiplicity of infection (moi) of 6∶1 with *L. amazonensis* transfectants containing pXG-GFP+. Following overnight infection, any unbound parasites were removed by washing twice with warm (37°C) PBS. Cultures were than incubated at 35°C for 10–15 days in RPMI1640 medium/10% FBS in the presence or absence of 35 µM peptide. Viable macrophages were analyzed by fluorescence microscopy to estimate the number of amastigotes per cell 10–15 days post infection. For increased accuracy, trypsinized macrophages were subjected to flow cytometry using a Becton Dickinson FACS Calibur. The percent of fluorescent macrophages, and the median fluorescence intensity were recorded.

### Immunoblotting

Immunoblotting was performed using standard protocols, as previously described, using either rabbit polyclonal anti-GFP (ab290, Abcam, Inc., Cambridge, MA) at a dilution of 1∶2000 for *L. amazonensis*, or with rabbit polyclonal antibody 2638 against EBNA1 [78] as a primary antibody. Immunoblots of nuclear and cytoplasmic fractions, prepared as previously described [79] from *L. amazonensis* control and pXGLamAT-GFP transfectants were probed with anti-GFP and anti-histone H3 (ab12079, Abcam, Inc., Cambridge, MA) at 1/500. For chemiluminescent detection, secondary goat anti-rabbit horse-radish peroxidase-conjugate (ab290 and antibody 2638) and goat anti-mouse horse-radish peroxidase-conjugate (ab12079) were used at 1∶5000.

### Evaluation of luciferase expression and plasmid replication & partitioning

Mammalian cell lines 293/LamAT-DBD or 293/HMGA1a-DBD were transfected with AGP53, a luciferase expression plasmid containing FR, and expressing luciferase under the control of a minimal HSV-1 TK promoter [49]. Luciferase expression was examined as a function of time as described previously, under conditions where the transfected cells were allowed to divide over the course of the experiment [56]. Transfections were normalized using a co-transfected GFP-expressing plasmid as described previously [49]. To examine the ability of LamAT-DBD to support licensed replication of an *oriP* replicon, plasmid AGP74, described previously [49] was transfected into 293/LamAT-DBD, or 293/HMGA1a-DBD cells. 48 hours post-transfection, cells were placed under puromycin selection for three weeks. A Hirt extract was performed on selected cells, and the level of episomal plasmid AGP74 was evaluated by Southern blot, using previously described procedures [49]. To evaluate plasmid partitioning, cells were transfected with AGP74 and plasmid 2145 that expresses GFP. 48 hours post-transfection, cells were subjected to flow cytometry to determine the percent of live-transfected cells (GFP-positive, propidium iodide-negative). 10^5^ transfected cells were plated on 10 cm dishes, and subjected to puromycin selection for three weeks as described previously [49]. Colonies were stained with methylene blue, and counted.

### Metaphase chromosome spread preparation and visualization

Metaphase chromosomes were isolated as described previously [19], [47], and visualized by indirect immunofluorescence using rabbit polyclonal antibody K67.3 that recognizes the DNA-binding region of EBNA1 as a primary antibody, and an AlexaFluor488-conjugated anti-rabbit secondary antibody. Chromosomes were counterstained with DAPI. Data was collected using a Zeiss AxioPhot microscope using a 100X objective, and 30 Z-sections of 0.05 µm each. Z-sections were deconvolved using the constrained iterative algorithm using Axiovision version 4.6. The data represents a maximal intensity projection (0° projection of the deconvolved stack).

### Indirect Immunofluorescence to determine sub-cellular localization

293, 293/LamAT-DBD and 293/HMGA1a-DBD cells were grown on cover-slips, fixed in 4% formaldehyde, and subjected to indirect immunofluorescence microscopy as described previously. The primary antibody used to detect LamAT-DBD and HMGA1a-DBD was either 2638 or K67.3, and an AlexaFluor488-conjugated secondary antibody was used. Nuclei were counter-stained with DAPI. *L. amazonensis* transfectants were subjected to indirect immunofluoresence as described above for mammalian cells, with the exception that parasites were grown in suspension and transferred to slides by cyto-centrifugation (1000 rpm, five minutes). Anti-GFP antibody (ab290) was used as the primary antibody, and Texas Red-conjugated secondary antibody was used for detection. DNA counterstaining was performed by treating with DAPI (0.5 µg/ml) for 90 to 120 seconds followed by washing with PBS. Image acquisition and processing was conducted as described for metaphase chromosomes for sub-cellular localization, with the exception that a 63X objective was used (NA 1.35).

### AT-richness analyses of the Chromosome 1 SSR

Sequences of all 15 SSRs were obtained from GenBank using the listed accession numbers. To examine AT-richness, sequences were scored using a matrix in which A or T were scored as 1, while G or C were scored as -1. The AT-distribution was calculated using 10 nucleotide tuples and a sliding window of one nucleotide. For example, using this matrix, a calulated value of 8 for a 10 nt tuple would indicate 9 A's or T's and 1 G or C. The number of uninterrupted AT-tracts of lengths between 2-10 nucleotides were calculated from tuples that ranged from 2–10 nucleotides and a sliding window of one nucleotide. Sequences were shuffled using Shuffle v1.02 released under GPLv2 by Edward Trager (University of Michigan). Pair-wise comparisons were used to compare the numbers of uninterrupted AT-tracts in shuffled sequences to values obtained for the SSR sequence. Statistical significance was calculated using the Wilcoxon rank-sum test.

## Supporting Information

Figure S1 Peptides corresponding to the first GR repeat and ATH from LamAT-Y bind AT-rich DNA specifically. (A) Peptides corresponding to amino-acids 121-155, P(121-155), and 290-323, P(290-323), for LamAT-Y were tested for their ability to bind poly(dA-dT) in the presence of excess poly(dA-dC)(dG-dT) as described in the Methods in nitrocellulose filter binding assays. The maximal binding measured by scintillation counting at saturating concentrations of each peptide (20 µM) was set at 100%. The binding curve for each peptide is indicated in the legend. (B) and (C) A titration of unlabeled poly(dA-dT), but not poly(dG-dC) effectively competes for the capacity of P(121-155) and P(290-323) to bind the labeled poly(dA-dT) probe. Competitors were added after peptide and probe were incubated for 20 minutes at room temperature. Peptide, probe and competitor were incubated for a further 10 minutes prior to filter binding. (D) The AT-rich DNA binding peptide netropsin displaces P(121-155) and P(290-323) from bound probe. A titration of netropsin was added after peptide and probe were incubated for 20 minutes at room temperature.
(TIF)

Figure S2 Expression analysis of LamAT-GFP and LamAT-Y. (A) mRNA levels of LamAT-GFP relative to the expression of GFP as evaluated by real-time PCR. Expression of LACK was used to normalize expression that was calculated by the ΔΔCt method as described in the Methods section. mRNA levels were quantified in four independent experiments (B) Relative expression of LamAT-GFP and GFP proteins. Extracts from untransfected, LamAT-GFP transfectants and GFP transfectants were examined by immunoblot using anti-GFP antisera. The number of cell equivalents used in each lane is indicated below each lane. The asterisk indicates an unknown Leishmania protein that is detected by the GFP-antisera. (C) The relative levels of LamAT 5′ mRNA ends detected in control cells or in LamAT-GFP transfectants evaluated by real-time PCR. In control, untransfected cells, these primers only detect LamAT-Y mRNA, while in LamAT-GFP transfectants, both LamAT-Y and LamAT-GFP transcripts are detected.
(TIF)

Figure S3 Analysis of AT distribution in chromosome 1 SSRs from 15 *Leishmania* species. The chromosome 1 SSR from *L. amazonensis*, and 14 other *Leishmania* was analyzed to determine the lengths and distribution of AT-runs. The AT distribution across the SSR using 10 nt tuples and a sliding window of 1 nt, scoring A or T as 1, and G or C as -1. These values were then plotted as a histogram (x axis = consecutive 10 nt tuples; y axis = score for each tuple). *Leishmania* species represented are as follows: *L. amazonensis* (DQ522035), *L. aethiopica* (DQ522034), *L. sp* MAR1/LEM2494 (DQ522036), *L. arabica* (DQ522037), *L. donovani archibaldi* (DQ522038), *L. braziliensis* (DQ522039), *L. donovani* (DQ522040), *L. enriettii* (DQ522041), *L. guyanensis* (DQ522042), *L. infantum* (DQ522043), *L. major* (DQ522044), *L. peruviana* (DQ522045), *L. tarentolae* (DQ522046), *L. tropica* (DQ522047), *L. turanica* (DQ522048).
(TIF)

Table S1 Identification of AT-hook containing proteins encoded by the *L. major* genome.
(PDF)

Table S2 Identification of L. major proteins containing three or more repeated GR or RG motif.
(PDF)

Table S3 Comparison of the effect of Dpep-GR1 on the number of macrophages and GFP+ amastigotes under control and Dpep-GR1 treated conditions.
(PDF)

Table S4 Statistical analysis examining the distribution of uninterrupted AT-tracts in the SSR sequence denoted by the solid red bar in Figure S2 for *L. amazonensis*.
(PDF)

Text S1 DNA sequence for LamAT-Y, the *L. amazonensis* ortholog of L. major LmjF06.0720.
(PDF)
