# Supplementary figures and images for: Molecular and Cellular Characterization of an AT-Hook Protein from Leishmania
Source: PLoS One. 2011 Jun 23;6(6):e21412. doi: 10.1371/journal.pone.0021412 (PMC3121789; doi:10.1371/journal.pone.0021412)

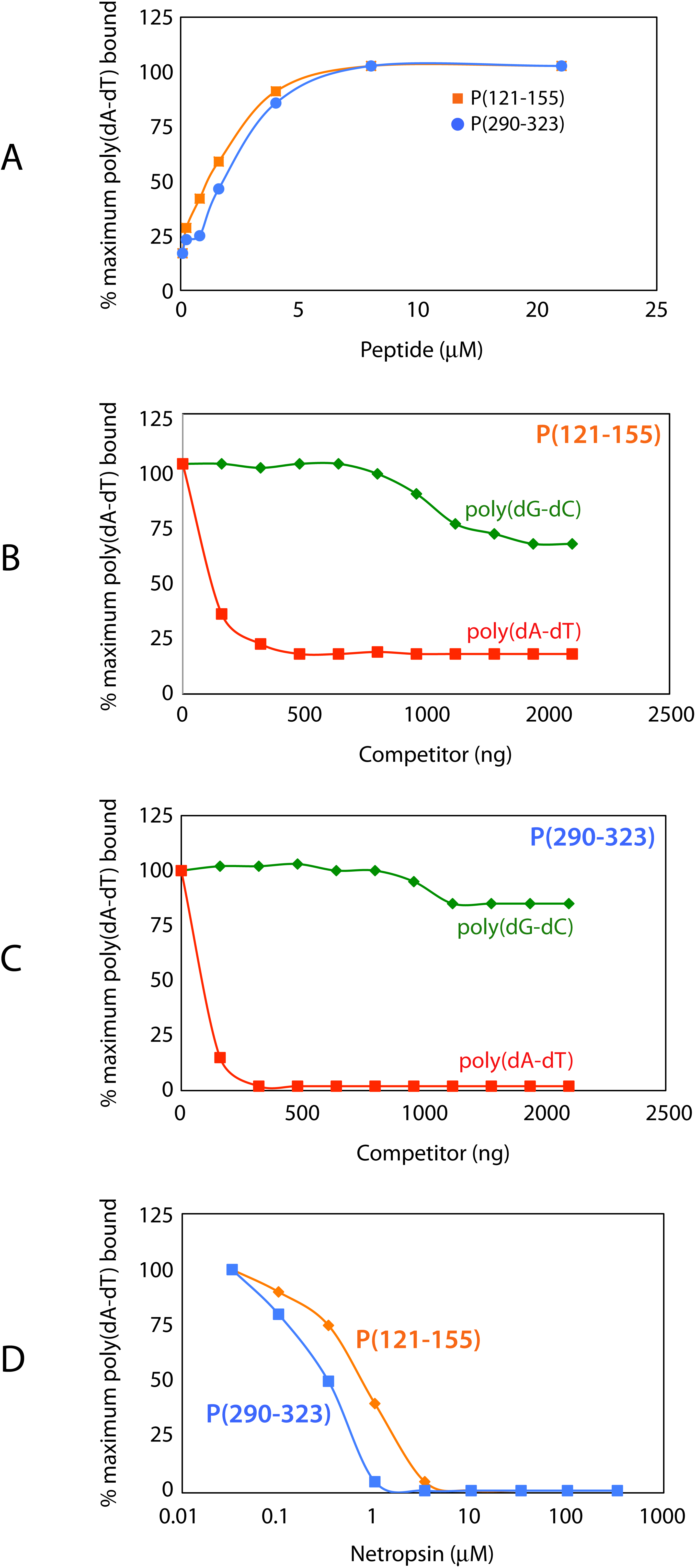

Supplement: Figure S1 — Peptides corresponding to the first GR repeat and ATH from LamAT-Y bind AT-rich DNA specifically. (A) Peptides corresponding to amino-acids 121-155, P(121-155), and 290-323, P(290-323), for LamAT-Y were tested for their ability to bind poly(dA-dT) in the presence of excess poly(dA-dC)(dG-dT) as described in the Methods in nitrocellulose filter binding assays. The maximal binding measured by scintillation counting at saturating concentrations of each peptide (20 µM) was set at 100%. The binding curve for each peptide is indicated in the legend. (B) and (C) A titration of unlabeled poly(dA-dT), but not poly(dG-dC) effectively competes for the capacity of P(121-155) and P(290-323) to bind the labeled poly(dA-dT) probe. Competitors were added after peptide and probe were incubated for 20 minutes at room temperature. Peptide, probe and competitor were incubated for a further 10 minutes prior to filter binding. (D) The AT-rich DNA binding peptide netropsin displaces P(121-155) and P(290-323) from bound probe. A titration of netropsin was added after peptide and probe were incubated for 20 minutes at room temperature. (TIF) [file pone.0021412.s001.tif]

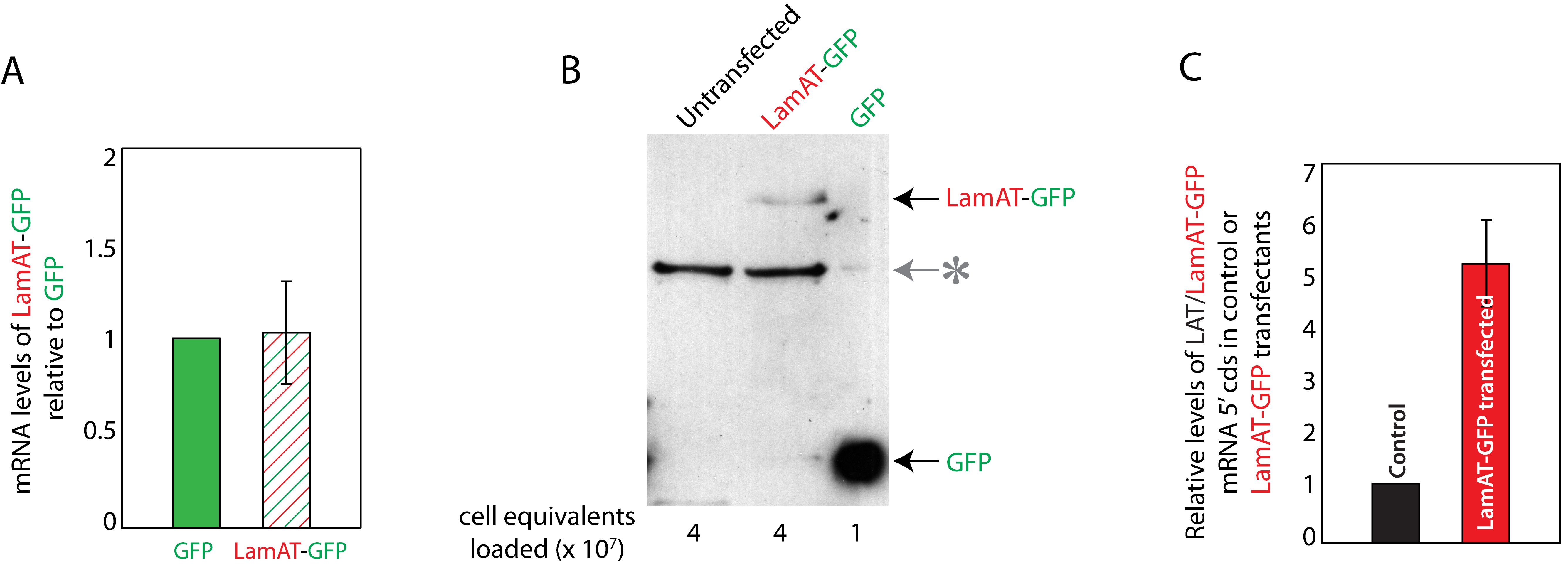

Supplement: Figure S2 — Expression analysis of LamAT-GFP and LamAT-Y. (A) mRNA levels of LamAT-GFP relative to the expression of GFP as evaluated by real-time PCR. Expression of LACK was used to normalize expression that was calculated by the ΔΔCt method as described in the Methods section. mRNA levels were quantified in four independent experiments (B) Relative expression of LamAT-GFP and GFP proteins. Extracts from untransfected, LamAT-GFP transfectants and GFP transfectants were examined by immunoblot using anti-GFP antisera. The number of cell equivalents used in each lane is indicated below each lane. The asterisk indicates an unknown Leishmania protein that is detected by the GFP-antisera. (C) The relative levels of LamAT 5′ mRNA ends detected in control cells or in LamAT-GFP transfectants evaluated by real-time PCR. In control, untransfected cells, these primers only detect LamAT-Y mRNA, while in LamAT-GFP transfectants, both LamAT-Y and LamAT-GFP transcripts are detected. (TIF) [file pone.0021412.s002.tif]

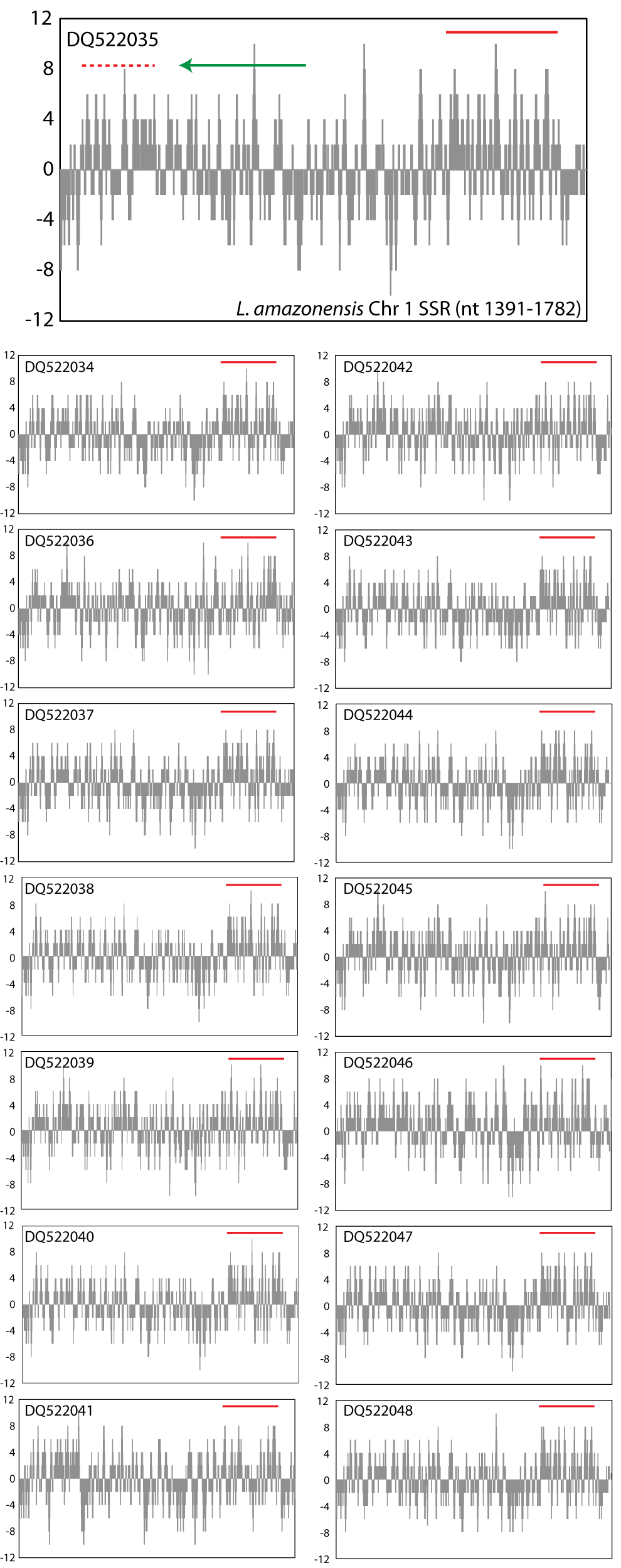

Supplement: Figure S3 — Analysis of AT distribution in chromosome 1 SSRs from 15 Leishmania species. The chromosome 1 SSR from L. amazonensis, and 14 other Leishmania was analyzed to determine the lengths and distribution of AT-runs. The AT distribution across the SSR using 10 nt tuples and a sliding window of 1 nt, scoring A or T as 1, and G or C as -1. These values were then plotted as a histogram (x axis = consecutive 10 nt tuples; y axis = score for each tuple). Leishmania species represented are as follows: L. amazonensis (DQ522035), L. aethiopica (DQ522034), L. sp MAR1/LEM2494 (DQ522036), L. arabica (DQ522037), L. donovani archibaldi (DQ522038), L. braziliensis (DQ522039), L. donovani (DQ522040), L. enriettii (DQ522041), L. guyanensis (DQ522042), L. infantum (DQ522043), L. major (DQ522044), L. peruviana (DQ522045), L. tarentolae (DQ522046), L. tropica (DQ522047), L. turanica (DQ522048). (TIF) [file pone.0021412.s003.tif]
