# Supplementary material for: Molecular and Cellular Characterization of an AT-Hook Protein from Leishmania
Source: PLoS One. 2011 Jun 23;6(6):e21412. doi: 10.1371/journal.pone.0021412 (PMC3121789; doi:10.1371/journal.pone.0021412)
Supplement: Table S1 — Identification of AT-hook containing proteins encoded by the L. major genome. (PDF) [file pone.0021412.s004.pdf]

Table S1. Identification of AT-hook motif containing proteins encoded by the *L. major* genome

Systematic Name	Number of AT-hook motifs	Additional Putative Domains Identified
LmjF06.0720	1	YEATS (ortholog of LamAT-Y)
LmjF31.0990	2	signal peptide
LmjF27.0430	1	none
LmjF35.2610	1	C-terminal membrane anchor, ubiqiutin-like domain
LmjF33.1100	2	CW-type Zinc finger
LmjF35.4050	1	Protein kinase
