# Supplementary material for: Molecular and Cellular Characterization of an AT-Hook Protein from Leishmania
Source: PLoS One. 2011 Jun 23;6(6):e21412. doi: 10.1371/journal.pone.0021412 (PMC3121789; doi:10.1371/journal.pone.0021412)
Supplement: Table S2 — Identification of L. major proteins containing three or more repeated GR or RG motif. (PDF) [file pone.0021412.s005.pdf]

Table S2. Identification of L. major proteins containing three or more repeated GR or RG motifs

Systematic Name	Number of repeated GR or RG motifs	Function or Additional Protein domains
LmjF19.0060, LmjF32.0450	3	40S ribosomal protein S2
LmjF35.1090	4	KRI-1 like family member
LmjF32.0400	3	RNA helicase
LmjF36.6980	3	Translation initiation factor 3, subunit 8
LmjF25.1980	3	Signal peptide
LmjF06.0720	7, 4	YEATS (ortholog of LamAT-Y)
LmjF32.1970	4	Rnase L inhibitor
LmjF29.1460	4	ATP binding, ATP transport
LmjF36.6570	9	unknown
LmjF36.5080	3, 4	unknown
LmjF33.0260	3	Signal peptide, RNA recognition motif
LmjF29.0680	3, 3	RNA binding protein
LmjF30.0760	4	Beta-catenin like
LmjF30.2610	4	RNA binding protein
LmjF29.0830	4, 3	One transmembrane domain
LmjF28.1780	5	DNA topo III
LmjF36.0980, LmjF36.0990	4, 3, 4	40S ribosomal protein S10
LmjF28.0825	3	RNA binding protein
LmjF28.0835	3	unknown
