# Supplementary material for: Molecular and Cellular Characterization of an AT-Hook Protein from Leishmania
Source: PLoS One. 2011 Jun 23;6(6):e21412. doi: 10.1371/journal.pone.0021412 (PMC3121789; doi:10.1371/journal.pone.0021412)
Supplement: Table S3 — Comparison of the effect of Dpep-GR1 on the number of macrophages and GFP+ amastigotes under control and Dpep-GR1 treated conditions. (PDF) [file pone.0021412.s006.pdf]

Table S3. Comparison of the effect of Dpep-GR1 on the number of macrophages and GFP+ amastigotes under control and Dpep-GR1 treated conditions

	Control	Dpep-GR1 Treated
Number of fields counted	14[1]	14
Macrophages/field	400.5 ± 125.6[2]	414.3 ± 36.3
GFP+ amastigotes/field	1.71 ± 3.04[3]	0.36 ± 0.63

[1]Infections were performed in chamber slides. DIC and direct fluorescence images were acquired as described in the methods. Fourteen images were acquired randomly from two independent infections. Acquired images were enumerated independently by two observers.

[2]Images were acquired at 400X using DIC optics. The numbers represent the mean number of macrophages ± SD.

[3]Direct fluorescence Images were acquired at 400X of the same fields used for DIC. The numbers represent the mean number of GFP+ amastigotes ± SD.
