# Supplementary material for: Molecular and Cellular Characterization of an AT-Hook Protein from Leishmania
Source: PLoS One. 2011 Jun 23;6(6):e21412. doi: 10.1371/journal.pone.0021412 (PMC3121789; doi:10.1371/journal.pone.0021412)
Supplement: Table S4 — Statistical analysis examining the distribution of uninterrupted AT-tracts in the SSR sequence denoted by the solid red bar in Figure S2 for L. amazonensis. (PDF) [file pone.0021412.s007.pdf]

Table S4: Statistical analysis examining the distribution of uninterrupted AT-tracts in the SSR sequence denoted by the solid red bar in Figure S2 for *L. amazonensis*

AT tract length (nt)	Predicted[1]	Observed[2]	Random (Average ± SD)[3]	*P*(Observed vs Random)[4]
2	133.8	132	132.7 ± 5.0	0.81
3	78.2	83	76.4 ± 8.6	4.9×10^{-5}
4	45.7	51	43.5 ± 9.0	3.6×10^{-5}
5	26.7	34	24.5 ± 8.0	1.2×10^{-7}
6	15.6	21	13.4 ± 6.4	5.2×10^{-7}
7	9.1	13	7.9 ± 4.9	2.3×10^{-8}
8	5.3	8	4.7 ± 4.2	3.3×10^{-7}
9	3.1	5	2.7 ± 3.4	2.0×10^{-6}
10	1.8	2	1.5 ± 2.8	0.02

[1]Predicted number of A+T tracts ranging from 2-10 nt for any 392 nt sequence that has an A+T content of 58.42%

[2]The number of contiguous A+T tracts ranging from 2-10 nt for the 392 3' AT-rich region from the chromosome 1 SSR (nt 1391-1782 of Genbank accession number DQ522035)

[3]A+T tracts ranging from 2-10 nt were enumerated in 30 randomly shuffled versions of the 3' AT-rich region from the chromosome 1 SSR. The average number and standard deviation for each tract length for the shuffled sequences is shown.

[4]P(two-sided) calculated using the Wilcoxon rank-sum test for pair-wise comparisons of observed versus randomly shuffled sequences
