# Supplementary material for: Molecular and Cellular Characterization of an AT-Hook Protein from Leishmania
Source: PLoS One. 2011 Jun 23;6(6):e21412. doi: 10.1371/journal.pone.0021412 (PMC3121789; doi:10.1371/journal.pone.0021412)
Supplement: Text S1 — DNA sequence for LamAT-Y, the L. amazonensis ortholog of L. major LmjF06.0720. (PDF) [file pone.0021412.s008.pdf]

```
LOCUS       LamAT-Y                 5265 bp    DNA     linear   INV 02-FEB-2011
DEFINITION  L. amazonensis ortholog of L. major gene LmjF06.0720.
ACCESSION   LamAT-Y
VERSION
KEYWORDS    .
SOURCE      Leishmania amazonensis
  ORGANISM  Leishmania amazonensis
            Eukaryota; Euglenozoa; Kinetoplastida; Trypanosomatidae;
            Leishmania.
REFERENCE   1  (bases 1 to 5265)
  AUTHORS   Kelly,B.L., Aiyar,A. and Singh,G.
  TITLE     Molecular and cellular characterization of an AT-hook protein from
            Leishmania
  JOURNAL   Unpublished
REFERENCE   2  (bases 1 to 5265)
  AUTHORS   Kelly,B.L., Aiyar,A. and Singh,G.
  TITLE     Direct Submission
  JOURNAL   Submitted (02-FEB-2011) Microbiology, Immunology and Parasitology,
            LSU Health Sciences Center, 1901 Perdido Street, New Orleans, LA
            70112, USA
COMMENT     Bankit Comment: TOTAL # OF SEQS:1.
FEATURES             Location/Qualifiers
     source          1..5265
                     /organism="Leishmania amazonensis"
                     /mol_type="genomic DNA"
                     /db_xref="taxon:5659"
     CDS             1..5262
                     /note="L. amazonensis ortholog of L. major LmjF06.0720.
                     This protein has three AT-rich DNA binding motifs and a
                     YEATS domain."
                     /codon_start=1
                     /product="LamAT-Y"
                     /translation="MYHPLPHGGGGSSKSVELHVDGRVSQVSLQAAAHATVADLLSTA
                     VAASSSTIPAKAPSPRELARGFACTDTAGNRVDLSALALSVPSQLMVTQTATAGSGGG
                     GSASVVSSGAGRGGGSGRSALSTSAPGRGRGRGRGRGRGGGRGRGGASAAAKAQTA
                     ADDEEDGDDDVDAEDGDGGEEVESGGATAEEDESGSNESDVPSEGYSSDDEDSSKVED
                     SDSSDSSLTTASTSTTTTRSRGDRGGATTSAGRGRGRGRSGTGRAASSRGSSAPPTPT
                     AASTTTARRTHRRSASGSPLLFTPDGLPIKRKRGRPPKSQQLALSNTAAAAAAAADAA
                     AASSGTMSTTVGEKPPRRTRRSVKEAPTEAAAEEEKPRRGGRRRRADAESEELKGQQR
                     ASASCKVMGGSSAVPVAPVDDVAGTLGGSGRRGGWRRGGGDAVTKRSRSPSPPSQLSG
                     GTSNTTTVAAPLFTASAVIVTAVDGTGGGCATPLSPGALTALVSGAGTDAAAATSAPG
                     ARTITAFEQQQQQEQLLRQQRHRVRLTYNTFYPSTTLHAAVPATTGTYGGSGTGGGTA
                     GARVHQAHSATAAAPLEDPLARPMRPRYVWNGGDRHQQHSLSSAAPLSPADNVQHGGR
                     GVPMTFREAWATLSSCLSSTTHCRGASSSASASPAIVDPLQHTASASQLYMQAQRIVP
                     SWRAEQHRTPSMLLSHPYSPLLMPGLRVYQHPLPLLTSTPAPLAAPADANTATPRRTD
                     RLSAVKVEVADEESDAGSRHRRHHQRGAAPHTTSVVAAASGAERDSSRVHCYPNQTLA
                     VPVVVGGCVQLINPESKDKSHQWTVYVRGLWNGHPEEVAGANARPPLSSHVQQLRAFP
                     SSPSSTPSALANSSRGSLGDSSATADRGGGGGAAAAALGAVVDSQTSVFSASTASPDD
                     YLSDFIDKVVFLLDESFVPCVRTVSSAPFELTEVGWGEFIVSMHVYLKVPVHTSASRR
                     AQGQLLEYFCGFNGRTALQTCDGRGTKPHDPVSTYVTGGVRGPSHLCNALTTPPPSTS
                     GTVPPTPALSLQELRHGCRYDSLLDINSNSGARATYYRGPYLPVHLVTCDATSSSASS
                     QLSSSSSDSDVDGGGGSDGARGRSGSEVSICGGSPTDSAHALSFPIKSEVSGDASFSS
                     SRSGSATPSPAPAPQHHANLSSQQHEARMGGPTTAHTSSSSIADGQPLAGIAAGVTDA
                     SSPSARPPPLPVPTVGRGTGRRPHRFGTSRGRSGTPRSPANSGAPSAAGGGGVHRTFT
                     EVHVGHGSNVVVLQHLLRFSHRPRYAAAIPPARDPRTQGAHPELLGYTMVAEPVVTEQ
                     YDELVIPLEPFVAVAERMRSQASRRWQTTQRTGGEASLKKPDAASVEAKLHHLLAASS
                     SLEGHLRATLRRQLSLMCDDSPSQSLHTLSPLSNSEAQALGHPGGISCWPHVLDYGNG
                     IERDTSYTAAYLASIATSAEMEGWLSQALGARRAALFGSPAASCAWVTTVDDAEDLQP
                     QRHGEAAGGCKTEGEGCDGVAEQIPGQVAIPSLIPNVATSGTWMTLLEYDMASALLRL
                     AAGASVVTAHDAFFQVVMPRADDIGAAVWCSLHEGNHAALSRANPVRPFPSDATGAAT
                     LKSEPVFWRTSELQQDTKKGAAVRMEQVHAEEECGGDESLSAPSAAALSRGYPLCSLR
                     PGDTGIAALRNVLCSNKGVGSVSAAHHPNQDAFMASLLRDGQNRGSFGHDSDVAQLLT
                     WKAALEDAIDTMRVEAARRQATAVMEEL*"
BASE COUNT      888 a   1780 c   1781 g    816 t
ORIGIN
        1 atgtaccatc ctctcccgca cgggggtggc ggcagctcga agagtgttga gttgcatgtg
       61 gacggccgcg tctcgcaggt ctccctgcag gcggcggcac acgccaccgt ggcggatctt
      121 ctctcgactg ccgtcgcagc ctcatcgtca accatcccgg caaaggcgcc gtcaccgcgg
      181 gagttggcga gggggtttgc ctgcaccgac acggcgggca acagggtcga cctgagcgct
      241 ctggcgctct ccgtcccgtc acagctgatg gtgacgcaaa ccgcgactgc cggcagcggt
      301 ggtggcggta gcgctagcgt ggtatcgagc ggcgccggcc gtggcggcgg ctctggccgc
      361 tcagcgttgt caacgtcggc ccctggccgt gggcgcggcc gcggtagggg gcgcgggcgt
      421 gggcgaggcg gcgggcgggg acgcggtggt gcctccgctg ctgccaaggc tcagaccgcg
      481 gccgatgacg aggaggatgg cgacgacgat gttgacgccg aggacggaga tggcggcgaa
```

541 gaggtcgagt ccggcggtgc cacggcagag gaagacgaga gtggcagcaa cgagtccgac
601 gtaccgtcgg agggctacag ctccgacgac gaggacagct ctaaggtgga ggacagcgac
661 agctccgact cctcgctgac cacagcgagc acaagcacca ccacgacacg ctcacggggc
721 gaccgcggcg gagcgaccac aagtgccggt cgtgggcgtg gacgtggtcg cagcggcact
781 ggtcgagccg cgtcttcgcg aggctcctcg gcgcccccca cgccgacagc ggcgagcacc
841 acaactgcgc gacgcaccca cagacgcagt gcctcgggat cgccgctgct cttcacccct
901 gacgggctac ccatcaagcg caaacgcggg cgaccgccaa agagccagca gctggcgctt
961 tcgaacaccg ctgccgctgc cgctgctgct gctgacgccg cggcggcatc gagcggcacc
1021 atgagcacca ccgttggtga gaagcctccg cggcggacgc ggcggagcgt caaggaggcg
1081 ccaactgagg cggcggcgga ggaggaaaag ccacgccggg gtggtcggcg ccgtcgcgcc
1141 gacgcggagt cagaggagct gaagggtcaa cagcgcgcga gtgccagttg caaagtcatg
1201 ggcggaagct ctgccgtccc agtcgcgcca gttgacgacg ttgccggcac tcttggtggc
1261 agcggccgac ggggtggatg gcggaggggt ggcggtgacg cggtcactaa gcgcagtcgg
1321 tcgccgtcac cgccttcaca gttgtctgga ggcaccagca acaccaccac cgtggcggcc
1381 ccgctattta ctgcgtccgc tgtgatcgtg accgctgtcg atggcacggg cggcggatgc
1441 gccactccgc tttccccagg cgccctcacc gcattggtga gcggtgcggg cacggatgcg
1501 gccgctgcga cgtctgctcc tggcgctcgc accattaccg ccttcgaaca gcagcagcag
1561 caggagcagc tgctacgtca gcagcgccac cgtgtgcgcc ttacgtacaa caccttctat
1621 ccatcaacca ctttgcatgc tgcggtgcca gccaccacgg gcacctacgg aggtagcggc
1681 acgggcgggg gcaccgcggg ggcacgcgtg catcaggcgc acagcgccac cgctgcagca
1741 ccgctggagg accctttggc gaggccgatg aggccgcgct acgtgtggaa cggcggagac
1801 cgccaccagc agcatagctt gtcctccgcg gcgccattgt cgcctgccga caacgtgcaa
1861 catggcggtc gcggcgtgcc gatgactttc agagaggcgt gggccacgct gtcttcttgc
1921 ctcagcagca ctacccactg ccgaggcgct tcatcctctg cgagcgcgtc ccccgcgatc
1981 gtggaccccc tccaacacac ggcgagtgca agccagcttt acatgcaggc tcagcggatc
2041 gtgccctcgt ggcgtgccga gcagcaccgc acgccgtcaa tgttgcttag ccacccctac
2101 agcccgctgt tgatgcctgg actgcgcgtg tatcagcacc cgctgccgct gctgacgtcg
2161 acacccgcac ctctcgcagc gccggcggac gcgaacacgg cgacgccccg caggactgat
2221 cggctgagtg ccgtaaaggt ggaggtggcg gacgaggaaa gcgacgcggg gtcgcgtcat
2281 cgccgccatc atcaacgtgg tgctgcaccg cacacgacat cggtggtggc cgctgcgtcc
2341 ggtgccgagc gcgacagcag ccgcgtgcac tgctatccga accagacgct ggctgttccc
2401 gtggtcgtcg gcggctgcgt gcagctcatc aacccggaga gcaaggacaa gagccaccaa
2461 tggacggtgt acgtgcgcgg gttgtggaac ggccatccgg aggaggttgc cggcgcgaat
2521 gcgcggccac ccttatcatc gcacgtgcag cagctgaggg cattcccttc gtcgccgtcc
2581 tctacaccat ctgctctggc gaacagctcg cgcggcagcc tcggcgacag cagcgcaact
2641 gccgaccgtg gcggtggcgg tggcgctgct gctgctgccc ttggcgcggt ggtcgattcg
2701 cagacctccg tcttcagcgc ctcaaccgcc tcgccggacg actacctgtc ggacttcatt
2761 gacaaggtgg tgttcctgct ggacgagagc ttcgtgccgt gtgtgcgcac cgtctcctcg
2821 gctccgtttg agctgacgga ggtggggtgg ggtgagttta ttgtgtccat gcacgtgtac
2881 ctgaaggtgc cggtgcacac cagtgcctcc cggcgggcac aggggcagct cctcgagtac
2941 ttctgcggct tcaacggccg cacggcactg cagacgtgcg acggtcgtgg cacgaagccg
3001 cacgaccccg tctcgacgta cgtcaccggc ggcgtgcgtg ggccgagcca cttgtgcaat
3061 gcgctgacga cgccgccgcc gagcacgtcg ggcactgtac cgcccacccc ggcgctgtcg
3121 ctgcaggagc tgcgccacgg gtgtcgctac gacagcttgc tagacatcaa ctcgaacagc
3181 ggggcgcgcg cgacctacta ccgtggtcca tacctgccgg tgcatttggt gacatgcgac
3241 gcgacgtctt cctcagcctc ctcgcagttg tcatcgtcat ccagtgacag cgacgtggat
3301 ggtggcggtg gtagtgacgg cgcacgcggc cgcagcggca gcgaggtgtc catctgtggt
3361 ggcagtccca cggacagcgc ccacgcactc tcgttcccca tcaagtcaga ggtaagcggt
3421 gacgcatcgt tctcctcttc ccgcagcgga agcgccactc ccagtccggc gccagcgcct
3481 cagcatcacg cgaacttgtc ctcgcagcag cacgaggcga ggatgggtgg gccaaccacc
3541 gcacacacca gcagcagcag catcgctgat gggcagccgc tcgcgggcat cgccgctggc
3601 gtgacggacg cctcttcgcc gagcgcgcga ccgccaccgc tgccggtgcc gactgtcggt
3661 cgcggcaccg gccggcgccc tcacaggttt ggcacttcgc gagggcgcag tggtacgcca
3721 cgcagccctg ccaactcagg cgctcccagc gctgcaggtg gcggcggcgt gcaccgcacc
3781 ttcacagagg tgcacgtcgg ccacggcagc aacgtggtgg tgctgcagca cctgctgcgc
3841 tttagtcacc gtcctcgcta cgcagccgcg attccgccag cgcgagaccc gcgcacgcag
3901 ggagcgcacc cagagctgct gggctacacg atggtggctg agcccgtcgt cacggagcag
3961 tatgacgagc tcgtcatccc gctggagcct ttcgttgctg tggcggagcg gatgcgcagt
4021 caggccagcc ggcgatggca gacgacgcag cgaacgggtg gcgaagcatc gctgaagaag
4081 cctgacgccg cgagtgtcga ggcgaagctg caccatctgc tggcagcatc gagcagtctc
4141 gagggccacc tgcgcgccac attgcgtcgc cagctctcct tgatgtgcga tgactcgcca
4201 tcgcagtcgc tccacacgct gtcgccactc tccaacagtg aggctcaggc gctcgggcac
4261 ccgggcggca tctcttgctg gccgcacgtc ctcgactacg gcaacggcat cgagcgcgac
4321 acatcctaca ccgctgccta cctcgcgagc atcgcgacga gcgcagagat ggaggggtgg
4381 ttgtcacaag cactcggggc acggcgggcg gcgctgtttg gctcgccagc ggcgtcttgt
4441 gcctgggtca ccaccgtcga cgacgctgaa gacctgcagc cgcagcgtca cggcgaggca
4501 gcgggagggt gtaagacgga gggggagggc tgcgatggcg tggcagagca aatacccggc
4561 caagtagcta ttccatccct catcccaaat gtggccacct ccgggacgtg gatgaccttg
4621 ctggagtacg acatggcgag tgcgctcttg cgcctcgccg ccggtgcctc cgtggtgacg
4681 gcacacgatg ccttcttcca ggtcgttatg ccgcgcgctg acgacattgg cgcagccgtc
4741 tggtgcagcc tccacgaagg aaatcacgca gcactaagtc gcgcgaatcc ggtccgaccc
4801 ttcccttcgg atgccactgg cgccgccacg ctcaagtcgg agccagtatt ctggcgcacc
4861 tcagaactgc agcaggacac caagaaaggc gcagccgtgc gaatggaaca ggtgcatgcc
4921 gaggaggagt gtggtggtga cgagtcgctg tctgcgccct cggcggcagc gctatctcgc

```
     4981 ggatatcccc tctgctccct gcgccccggt gacaccggca ttgccgcgct gcgcaacgtg
     5041 ctctgctcga acaaaggcgt cggctccgtc agcgccgctc accacccaaa ccaggatgcg
     5101 ttcatggcgt cgctgctccg tgacggccaa aaccgcggca gcttcggcca cgacagcgat
     5161 gtggcgcagc tgctgacatg gaaggcagcg ctagaggacg ccatcgacac catgcgcgtc
     5221 gaagcagcgc gccggcaggc cactgctgtc atggaggagc tgtaa
//
```
